# The role of climate and environmental variables in structuring bird assemblages in the Seasonally Dry Tropical Forests (SDTFs)

**DOI:** 10.1371/journal.pone.0176066

**Published:** 2017-04-25

**Authors:** Gabriela Silva Ribeiro Gonçalves, Pablo Vieira Cerqueira, Leandro Schlemmer Brasil, Marcos Pérsio Dantas Santos

**Affiliations:** 1 Curso de Pós-Graduação de Zoologia, Universidade Federal do Pará / Museu Paraense Emílio Goeldi, Terra Firme, Belém, Pará, Brazil; 2 Universidade Federal do Pará, Instituto de Ciências Biológicas, Laboratório de Ecologia e Zoologia de Vertebrados, Belém, Pará, Brazil; Shanxi University, CHINA

## Abstract

Understanding the processes that influence species diversity is still a challenge in ecological studies. However, there are two main theories to discuss this topic, the niche theory and the neutral theory. Our objective was to understand the importance of environmental and spatial processes in structuring bird communities within the hydrological seasons in dry forest areas in northeastern Brazil. The study was conducted in two National Parks, the Serra da Capivara and Serra das Confusões National Parks, where 36 areas were sampled in different seasons (dry, dry/rainy transition, rainy, rainy/dry transition), in 2012 and 2013. We found with our results that bird species richness is higher in the rainy season and lower during the dry season, indicating a strong influence of seasonality, a pattern also found for environmental heterogeneity. Richness was explained by local environmental factors, while species composition was explained by environmental and spatial factors. The environmental factors were more important in explaining variations in composition. Climate change predictions have currently pointed out frequent drought events and a rise in global temperature by 2050, which would lead to changes in species behavior and to increasing desertification in some regions, including the Caatinga. In addition, the high deforestation rates and the low level of representativeness of the Caatinga in the conservation units negatively affects bird communities. This scenario has demonstrated how climatic factors affect individuals, and, therefore, should be the starting point for conservation initiatives to be developed in xeric environments.

## Introduction

Understanding the processes that contribute to the establishment and diversity of species is still a challenge in ecological studies since there are many mechanisms and factors that may affect biological diversity [1–3]. This diversity is inserted in a dynamic and complex ecosystem, where factors such as predation [4,5], the Allee effect [6], intra and inter-specific interactions, competition for food [4,7], and environmental [8] and spatial factors [9] may affect its distribution and permanence.

Communities do not have well-defined boundaries and there is a slow and gradual turnover along a gradient in most situations [10,11]. Two main theories have been used as the basis for discussions on this topic—the niche [1] and neutral theories [12]. The niche theory posits that the main factors that affect species distribution are local environmental characteristics, where each species tolerate a set of biotic and abiotic factors that define its limit of occurrence [13]. The neutral theory postulates that composition is a result of stochastic factors along with dispersal limitation, where the combination of these factors can lead to communities with different characteristics [12,14,15].

Birds are organisms with high dispersal ability, that participate in many biological interactions and are usually highly faithful to specific habitats [16,17]. In addition, birds make up the most diverse group of terrestrial vertebrates, comprising species that are excellent environmental indicators that respond quickly to subtle changes in the environment [18]. Thus, understanding biotic and abiotic mechanisms that affect the distribution of bird species may play a key role in the discussion of the processes that contribute to the establishment and diversity of species.

A set of extremely severe environmental conditions associated with more extreme weather condition within Brazil are found in the Caatinga, the largest area of Seasonally Dry Tropical Forest (SDTF) located in northeastern region of Brazil [19,20]. The local biota in this type of environment is expected to have unique adaptations for local survival, making it an especially important region to study the interrelationships of biotic communities in xeric environments. Annual climatic variations affect the availability of food resources, nesting sites and vegetation structure [21], affecting community structure and bird species richness [21–23]. Seasonality between the dry and rainy seasons clearly affects the abundance of food resources, where rainy seasons have greater environmental complexity, and higher supply and availability of resources [22]. These factors directly affect composition, abundance and richness of bird communities [24–26].

Climate is a key environmental variable for the occurrence and abundance of birds in the landscape, in both time and/or space [16,27,28]. Variables such as humidity, solar radiation and air temperature affect the metabolic rate of animals and plants, producing different levels of physiological responses [29,30], thus influencing the egg hatching rates in birds, where high temperatures associated with low humidity ranges can lead to reduced reproductive success [31]. Therefore, this set of factors makes seasonality an important environmental filter for species distribution, resulting in changes in the composition and structure of communities [32].

Our objective is based on biological and on the aforementioned theoretical aspects, and consists on understanding the importance of environmental and spatial processes on richness and composition of bird communities among the hydrological seasons in areas of seasonally dry forests. We sought to answer the following questions: (1) Do richness and composition measures vary among hydrological seasons? (2) What factors (environmental or spatial) can explain the variation in richness and composition of bird species in dry forest areas?

## Materials and methods

### Study area and sampling design

**Fig 1:**
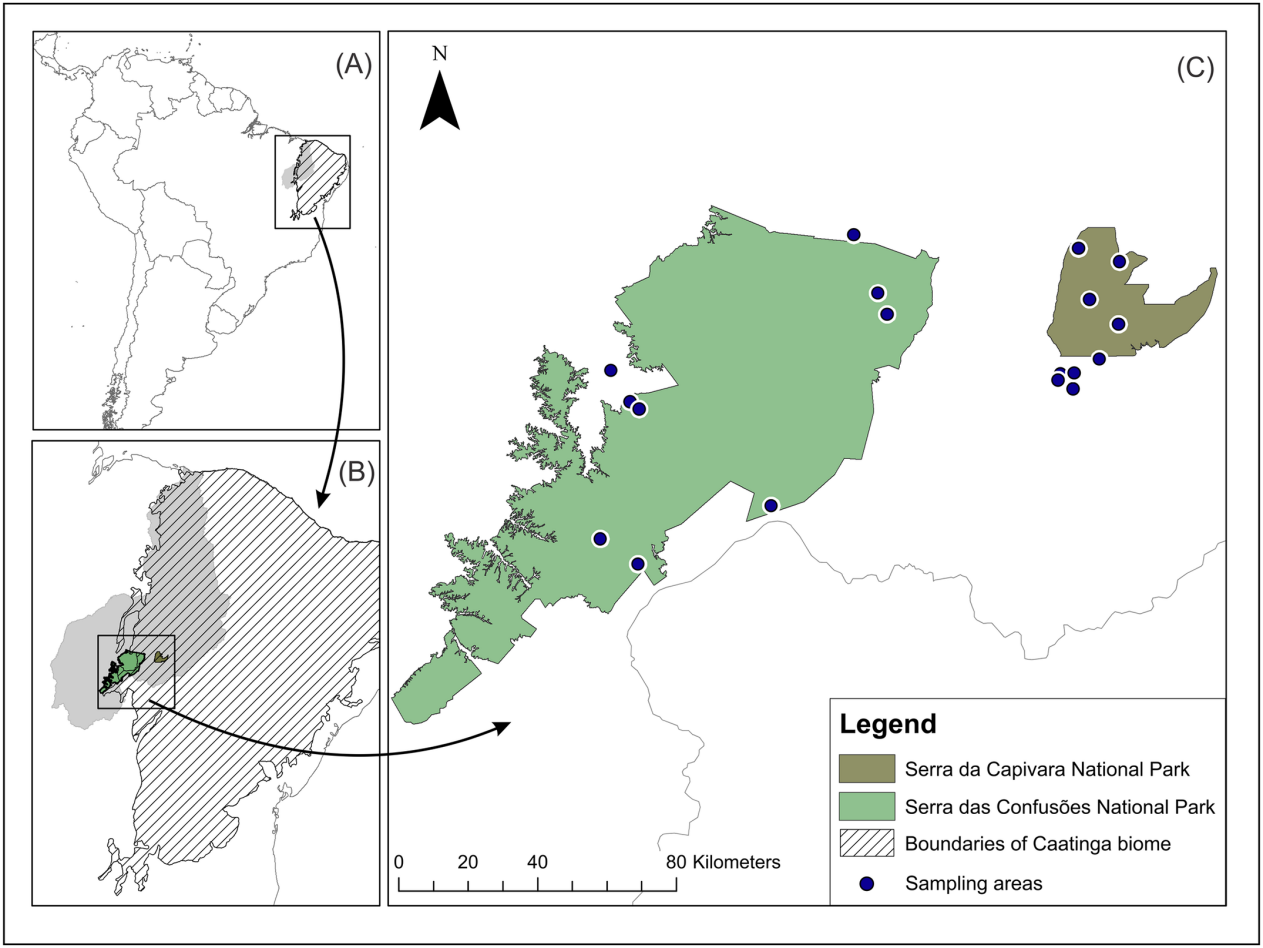
Map of the study area showing the location of sampling areas in the Serra da Capivara and Serra das Confusões National Parks. Blue circles indicate locations of the 18 sampling areas. A) Wide view of the location of study area in relation to South America, B) location of the study area within the state of Piauí and in the Caatinga biome, C) location of the sampling sites in the National Parks and vicinities. Shapefile of biome are provided by MMA (Ministério do Meio Ambiente do Brasil).

This study was carried out in two full protection Conservation Units, Serra da Capivara and Serra das Confusões National Park, both located within the Caatinga biome (Northeastern Brazil) and were considered priority areas for biodiversity conservation by Biodiversitas in 2000 and by the committee of "Evaluation and Priority Actions for Conservation of Caatinga Biodiversity" (Fig 1) [33].

The Serra da Capivara National Park, has 130 hectares and was created in 1979 with the main goal of protecting the rich archaeological heritage of the region [34]. This park is located in the southeastern state of Piauí (northeastern semiarid region), between two geological formations, with mountains, valleys and plains. The climate is transitional Tropical semi-arid and tropical sub-humid dry [35], and is defined as BSwh’ (high temperature and strong evaporation in the summer) according to the Köppen classification [36]. This region has an annual temperature of 28°C, June is the coldest month (minimum temperature of 12°C) and October the hottest month (maximum temperatures above 45°C). Rainfall is concentrated in from November to March, with an average annual rainfall of 689mm [37,38]. The vegetation is deciduous, where a drastic change in plant biomass may be observed along the year. Such changes in biomass strongly reduces in the peak of the dry season and gradually increases in the transition from the dry to the rainy season, when the maximum leaf cover is observed. The understory is abundant in the rainy season, disappearing completely in the dry season [38]. Emperaire [39] described eight different vegetation types for the Park. This park is covered mostly by a high shrubby vegetation of 6-10m in the central plateau, and other vegetation types, ranging from mesic forests in canyons to shrubs in rocky areas, composed mostly of cacti and bromeliads [39]. The park does not have a permanent watercourse, but several small natural reservoirs ("caldeirões").

The Serra das Confusões National Park, located in the southwestern state of Piauí, has a total area of 823,435.70 ha [40], and is located within the inter-plateau depressions of the semi-arid regions of Brazil and, is therefore, characterized by having a hot and dry climate determined by a markedly seasonal rainfall. In general, the rainy season begins in mid-November lasting until April, when the dry season begins, with average annual rainfall of 600 mm [40,41]. The whole area of this park is located within the sandstone plateau region (plateaus) and depressions of the Parnaíba Basin. The vegetation in the region of the Serra das Confusões National Park has a canopy of up to 6 meters, with three prevailing vegetation types: Arboreal, shrubby and arboreal/shrubby, and the presence of predominantly deciduous species (all the foliage of the vegetation is lost during the dry season), as well as understories of deciduous forests in riparian areas and in places where the weather conditions are more mesic [42].

We chose a sampling design that encompassed the topographic and vegetation variability of the study area to capture most of the environmental diversity of the parks. Accordingly, the canyon areas (wetter and forest-sized vegetation), the "chapadas" (top of the plateaus; vegetation is drier and composed of trees and shrubs), and, finally, areas undergoing an ecological regeneration process, here called “Caatinga under regeneration” were selected as the treatments. The area called “Caatinga under regeneration” are in fact areas that have been deforested in the past to be used in subsistence agriculture. These regeneration areas were selected based on the "resting" ages, which should be between 8 to 10 years. So we have three sampling areas per type of environment (canyon, "chapada" and Caatinga under regeneration) in each of the National parks studied. Thus, we selected 18 sampling areas (9 in each park and vicinities). Each sampling area (sampling unit) consists of a 3 km trail along which quantitative and qualitative data of the bird community were carried out, in addition to the environmental data.

Four sampling works were conducted in each sampling area considering the hydrological cycles as follows: in the dry season (July 15 to August 08, 2012), in the rainy season (February 03 to 22, 2013), rainy-dry transition (May 25 to June 18, 2013), and dry-rainy transition (December 09 to 23, 2013). Each of the four seasons will be treated in this article as a hydrocycle. None birds were captured or manipuled in this work and Instituto Chico Mendes de Conservação da Biodiversedade was permited this field work.

### Data collection

We conducted 15 fixed width (75m) 10-minute point counts per sampling area, located at 200 m-intervals to obtain the abundance, richness and composition of bird species [43,44]. Each sampling area was sampled during four hydrocycles, so the sampling effort for each area was 60 point counts or 600-minute census. The surveys were started always from early morning, near to sunrise, until the last point count and were not carried out on days with persistent rain and/or strong winds.

We used an adapted protocol for the study area to measure vegetation structure parameters of each site (based on Peck *et al*. [45]). This protocol aims to quantify the largest possible number of environmental variables to evaluate their effect on species composition and diversity. Vegetation data were collected in three plots established in each sampling area (3x3 m plots and spaced 500m from each other). These plots were set in the same tracks used for the point counts carried out for bird surveys. The quantitative variables measured were litter height (cm, measured using a ruler from the soil until the top of the leaf litter), temperature (°C) and humidity (%), both measured using a digital thermo-hygrometer. Three measures were taken in each plot and we used the mean of this values for the plot and the mean of the three plots for each site. The vegetation cover characteristics were measured through visual estimations of the following parameters: canopy (above 2 m), understory (from 0.5 to 2 m) and undergrowth (< 0.5 m). The number of large trees (Diameter at breast height, DBH> 15cm), number of small trees (DBH<15cm), woody shrubs and seedlings, herbs without woody stem and grass, bare soil or litter were counted to characterize the vegetation, all these were measured as categorical classes by percentage of occurrence in each plot. All variables of the protocol were categorized according to frequency of occurrence and then transformed into quantitative measures following Kaufman *et al*. [46].

An adaptation of the method proposed by Marsden *et al*. [47] was used to obtain understory and canopy complexity measures in the plots. These measures were determined from digital photos (taken using a Nikon camera; Coolpix S3300) taken 1.20 m above the soil vertically for canopy complexity and at 3 m distance from a white fabric (2x2 m) as background for understory complexity. The photos were converted to grayscale to enable calculating the percentage of white and black pixels in the image using the software ENVI 4.5 (ITT Visual Information Solutions, Boulder, CO, USA), we used the index of black pixels to represent the complexities and for each site was used the mean of index of the three plots.

The regional environmental variables, precipitation and temperature, were extracted from maps with a spatial resolution of 1km obtained from the Image Processing and Geoprocessing Laboratory of the Federal University of Goiás (LAPIG-UFG) (http://www.lapig.iesa.ufg.br/lapig). A regional temperature and precipitation map was obtained for each period of time where biological samples were collected (month/year).

### Data analysis

We used a 1st Order Jackknife estimator with the software EstimateS Win 7.5.0 to evaluate whether there was difference in richness between the Hydrocycles [48], using the confidence interval technique, where two groups are only considered different when the confidence interval of a group does not overlap the average of the other. We tested the difference in composition between the Hydrocycles using a Principal Coordinates Analysis (PCoA) with the transformed abundance data, using a Bray-Curtis distance matrix, and the Broken-Stick criteria for o select the axes [49]. An Analysis of Similarity (ANOSIM) was performed to check whether the groups formed by the PCoA were different based on species composition [50].

A Principal Component Analysis (PCA) was carried out to reduce environmental variables and determine which environmental variables can explain the environmental variation among Hydrocycles. Therefore, variables were standardized and the Broken-Stick criteria was used to choose the axes [49]. An analysis of Similarity (ANOSIM) was performed to check whether the groups formed by PCoA were different based on species composition [50].

We calculated spatial filters (PCNM) to understand the effects of the spatial conformation of sites [51,52], testing aspects of the neutral theory [12]. This approach may show the proportion of the explanation of the models that is related t*o the spatial distribution of sites, and is also used with environmental predictors minimizing biases related to spatial autocorrelation of sites, reducing the likelihood of type 1 error in the model results [53]. We extracted the values from the spatial cell where the sites were located using the function *extract* from the *raster* package [54] to obtain continuous numerical values for the regional environmental predictors (precipitation and temperature) for each site, in each hydrocycle. We used the function *pcnm* of the package *vegan* [55] to calculate the spatial filters. All these analysis (extract and pcnm) were carried out in the software R [56].

A Pearson correlation analysis between all environmental predictors was built to evaluate the effect of environmental variables on species richness and composition, first minimizing the potential problem of multicollinearity among the predictor variables, and reducing the number of predictor variables. When a variable was correlated ≥ 70% to one or several variables in the analysis, only one was retained. The criteria for choosing which variables were withdrawn or maintained in the set of predictors after the collinearity analysis followed what was established in the literature as important for the richness and composition of bird species. When a set of predictors were correlated, we maintained the variable we deemed more important following the literature criterion. A multiple regression was carried out [57] between the environmental variables and the PCNMs selected as the predictors, and the species richness of each sample unit as the response variable. Then, we carried out a Multivariate Distance Matrix regression—MDMR [58,59] using the same predictors used in the multiple regression to assess whether the environmental variables can explain the variation in species composition and which variables affect composition.

## Results

**Fig 2:**
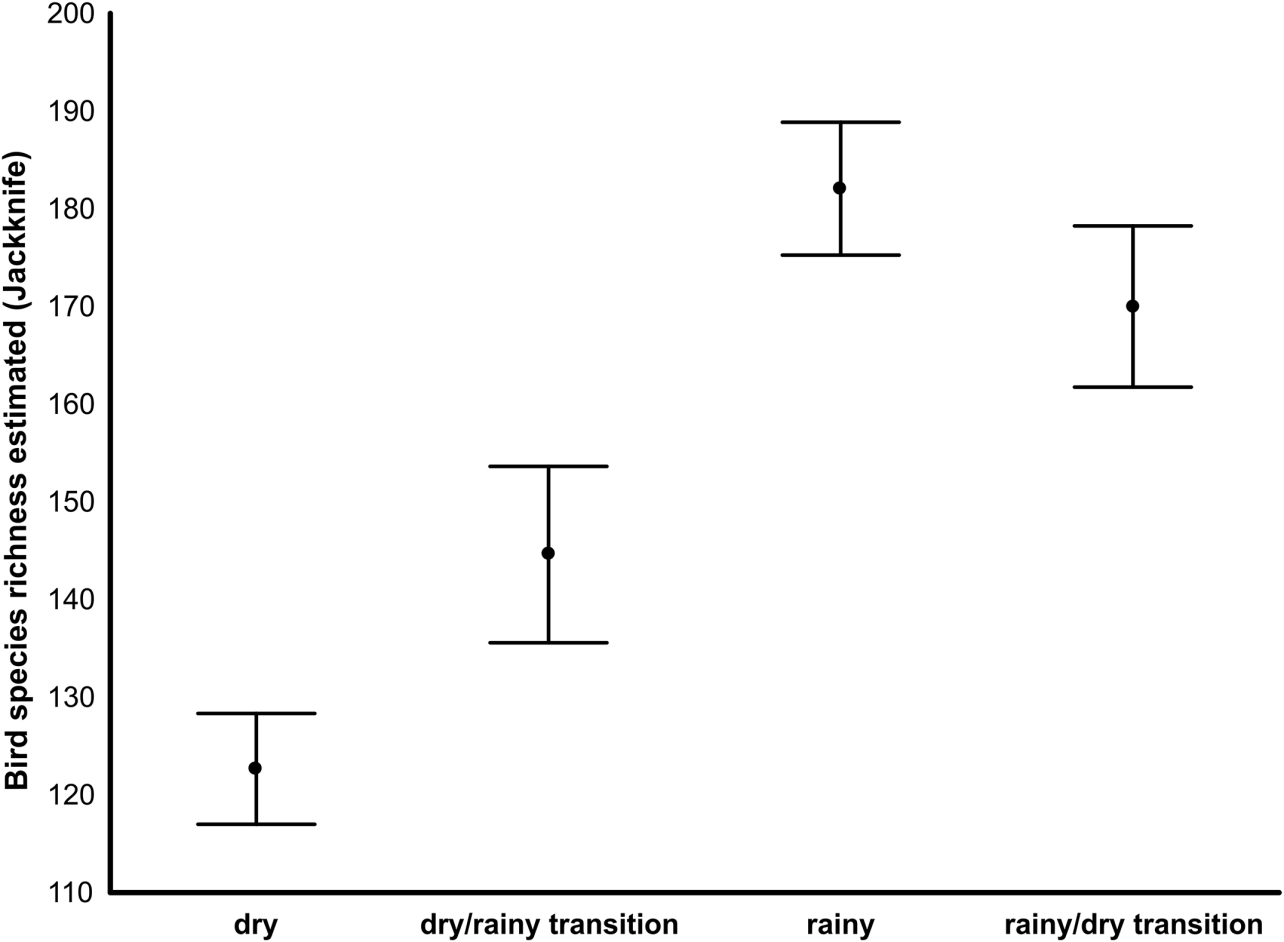
Bird species richness estimated by the 1st order Jackknife estimator in each hydrocycle. The survey was carried out in two areas of Caatinga, namely the Serra da Capivara and Serra das Confusões National Parks, state of Piauí, from 2012 to 2013. Error bars represent the 95% confidence interval of the mean.

A total of 177 bird species (see S1 Table for details) were recorded with an estimated richness of 215±4.76 (mean±confidence interval), of which 100 (122.67±5.67) were recorded in the dry season, 121 (144.61±9.03) in the dry/rainy transition, 149 (182.06±6.8) recorded in the rainy season and 136 (170±8.25) recorded in the rainy/dry transition. Thus we found a significant difference in species richness of birds among the hydrocycles, once the confidence intervals did not overlap the mean of the others, suggesting strong seasonal dynamics in bird communities of the Caatinga region (Fig 2).

**Fig 3:**
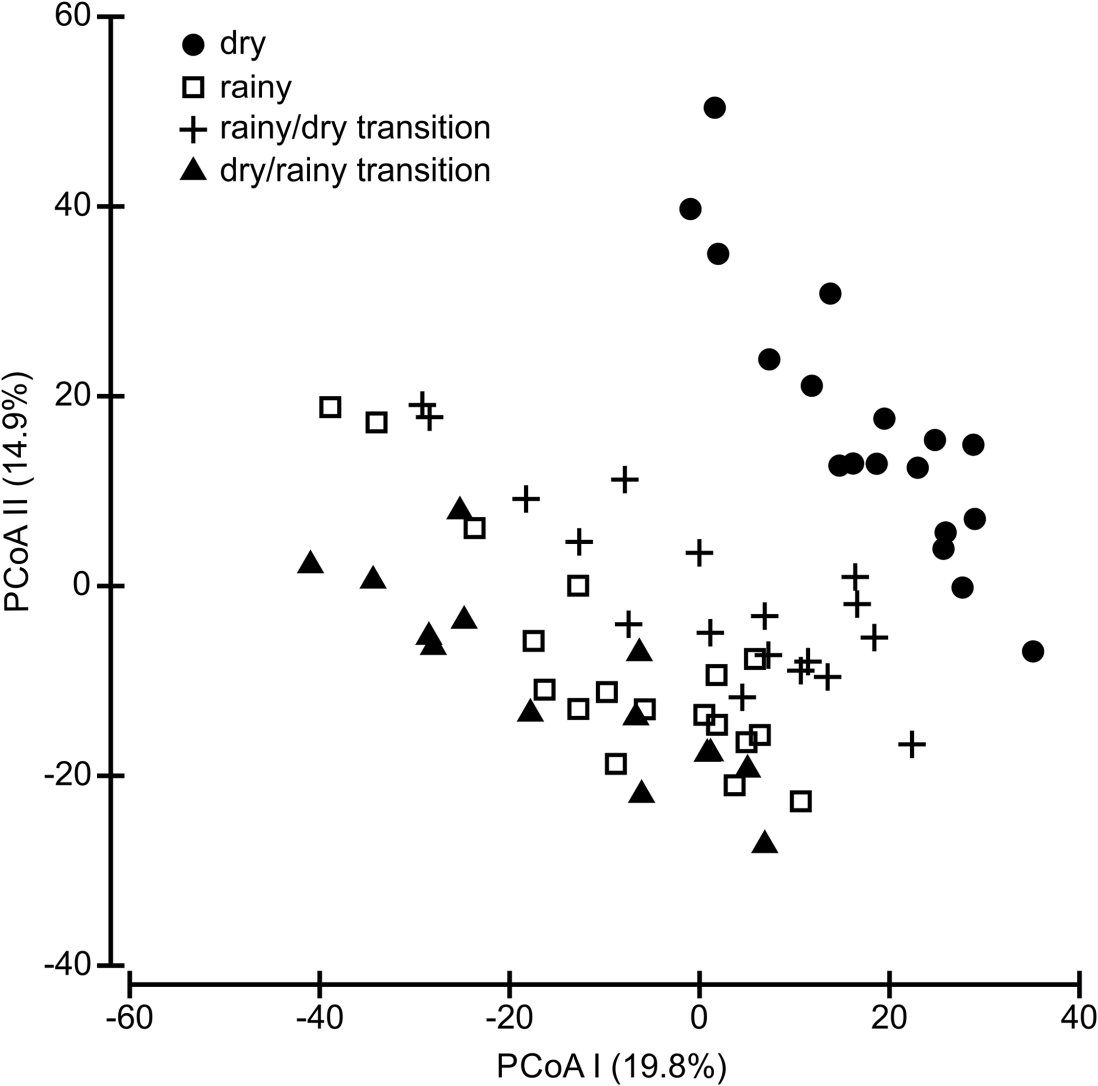
Principal Coordinates Analysis (PCoA) ordination of species composition of bird species among hydrocycles, using the Bray-Curtis distance matrix of transformed abundance data. Dry season are represented by circles, Rainy by squares, Rainy/dry transition by plus and Dry/rainy transition by triangles. Numbers in parentheses are the percentage of variance explained by each axis.

The ordination explained 34.7% of the community composition in the first two axes, 19.8% in the first and 14.9% in the second, which were selected according to the Broken-Stick criterion. The ordination partially separated communities by hydrocycles. In the first axis the composition of the dry season (right) and dry/rainy transition (left) were the most different, while the rainy and dry/rainy transition exhibited a greater overlap (Fig 3). The Anosim confirmed that the compositions differed among hydrocycles (R = 0.401; p = 0.001) and in the pairwise analysis, where the highest difference was recorded between the composition of the dry and dry/rainy seasons (R = 0.736; p = 0.001) and the lowest between the rainy and dry/rainy season (R = 0.137; p = 0.012) (Table 1).

**Table 1:** ANOSIM of the composition of bird species among hydrocycles in two areas of the Caatinga, namely the Serra da Capivara and Serra das Confusões National Parks, state of Piauí, Brazil.

Groups	R	p
dry, rainy	0.597	0.001
dry, rainy/dry	0.423	0.001
dry, dry/rainy transition	0.736	0.001
rainy, rainy/dry transition	0.185	0.001
rainy, dry/rainy transition	0.137	0.012
rainy/dry transition, dry/rainy transition	0.32	0.002

**Fig 4:**
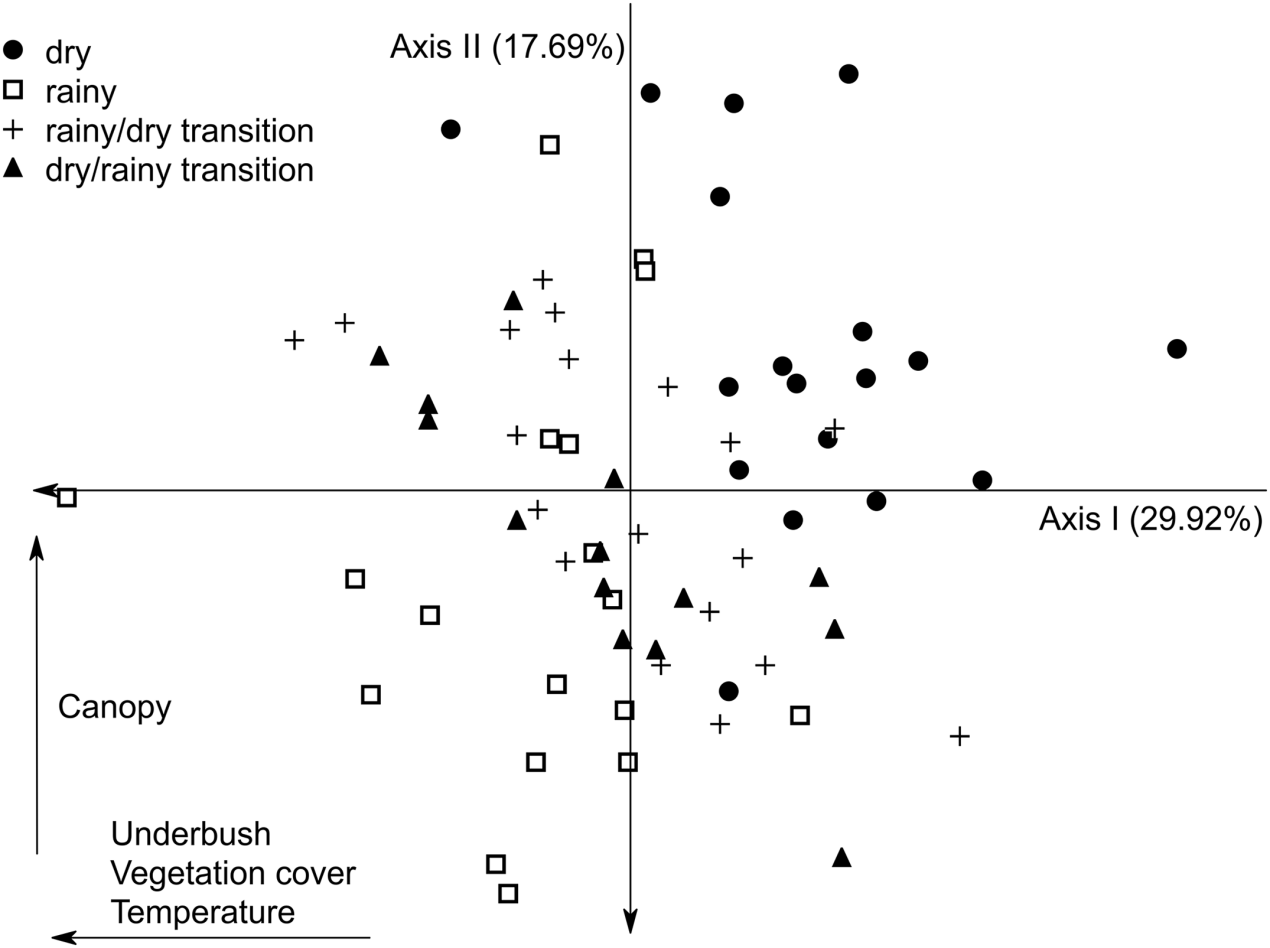
Principal Component Analysis (PCA) ordination for the environmental variables selected in the first and second axis (first axis: Underbush, vegetation cover, temperature and second axis: canopy). Dry season are represented by circles, Rainy by squares, Rainy/dry transition by plus and Dry/rainy transition by triangles. Numbers in parentheses are the percentage of variance explained by each axis.

The ordination made with the environmental variables reinforced the existence of a robust environmental difference among the four hydrocycles used in this study. The first two axes of the PCA chosen by the Broken-Stick criterion comprised 47.61% of the environmental variation, where the first axis explained 29.92% and the second 17.69%. There was a separation between the dry and rainy seasons in the first axis (horizontal) (Fig 4). However, there was no clear separation of the transitional groups, whereas temperature, underbrush and vegetation cover are the environmental variables that most contributed to this pattern, all negatively related to the axis. The second axis (vertical) distinguished the two seasons according to the canopy, where there was greater canopy amplitude in the rainy season than in dry season. The transitional season showed intermediate values for the variables, being different from the dry and rainy seasons, but not from each other. The ANOSIM in the pairwise analysis of the environmental data significantly separated the hydrocycles from each other (R = 0.603; p = 0.001) (Table 2), despite the difficulty in viewing groups in the PCA.

**Table 2:** ANOSIM with environmental data across hydrocycles, in two Caatinga areas, namely the Serra da Capivara and Serra das Confusões National Parks, state of Piauí, Brazil.

Groups	R	p
dry, rainy	1	0.001
dry, rainy/dry transition	0.374	0.001
dry, dry/rainy transition	0.496	0.001
rainy, rainy/dry transition	1	0.001
rainy, dry/rainy transition	1	0.001
rainy/dry transition, dry/rainy transition	0.265	0.001

The effect of the environmental variables on bird species richness was evaluated through a multiple regression. The model composed of the set of predictors explained 36% of the variation in species richness (R^2^ = 0.36, F = 3.65, p = 0.001). Observing the variables within the model individually, we found that vegetation cover and microclimate temperature were significant, where the vegetation cover had a negative slope and microclimate a positive slope regarding species richness (Table 3), indicating that species richness decreases in environments with less vegetation cover and increases with lower local temperature.

**Table 3:** Result multiple regression of species richness and environmental variables in Caatinga areas, in the Serra da Capivara and Serra das Confusões National Parks, state of Piauí, Brazil.

Variables	Coeff.	Std Coeff.	VIF	Std Error	t	P
Constant	39.915	0	0	25.566	1.561	0.124
Vegetation cover	-0.269	-0.24	1.258	0.131	-2.052	0.045
Microclimate temperature	0.289	0.317	1.741	0.126	2.3	0.025
Canopy complexity	0.622	0.052	1.265	1.406	0.442	0.66
Litter	6.413	0.125	1.231	5.922	1.083	0.283
Understory complexity	-0.094	-0.116	1.648	0.109	-0.862	0.392
Precipitation	0.015	0.159	2.283	0.015	1.006	0.319
Regional temperature	-0.274	-0.068	2.441	0.661	-0.415	0.68
PCNM1	-2.425	-0.027	1.278	10.72	-0.226	0.822
PCNM2	18.971	0.209	1.196	10.367	1.83	0.072

We analyzed which factors may explain the variation in species composition among the studied areas with the MDMR. We found that vegetation cover, microclimate temperature, canopy complexity, complexity of understory and precipitation positively affected species composition, and explained a total of 26% of the variation in species composition. The spatial predictors (PCNM1 and PCNM2) explained only 0.08% of species composition (Table 4).

**Table 4:** Result of the Multivariate Distance Matrix regression (MDMR) of species composition and environmental variables variables in Caatinga areas, in the Serra da Capivara and Serra das Confusões National Parks, state of Piauí, Brazil. Species composition was calculated using transformed abundance data and a Bray-Curtis distance matrix.

	Df	F.Model	R2	Pr(>F)
Vegetation cover	1	1.977	0.024	0.015
Microclimate temperature	1	4.563	0.055	0.001
Canopy complexity	1	4.259	0.051	0.001
Litter	1	1.336	0.016	0.157
Understory complexity	1	1.752	0.021	0.036
Precipitation	1	1.949	0.023	0.01
Regional temperature	1	1.144	0.014	0.274
PCNM1	1	3.551	0.043	0.001
PCNM2	1	3.634	0.044	0.001
Residuals	59		0.709	
Total	68		1	

## Discussion

We found in this study a greater bird species richness in the rainy season and lower in the dry season. This result shows a strong influence of hydrocycles on the bird community of the SDTF’s of the Caatinga, suggesting restrictive conditions caused by irregular rainfall [60,61]. These conditions can determine the success of some species, according to their adaptations to overcome the conditions imposed by the environment [62].

Some patterns of community structure can be strongly affected by the seasonal climatic events in environments with xeric vegetation, such as the SDTF’s. Such environments typically exhibit a short favorable season, which demands rapid development and reproduction from species. This is mainly due to the more limiting conditions of dry season, and more favorable in the rainy season when water supplies become more available and food resources such as fruits and arthropods, also increase in abundance [28,63]. Phenological studies conducted in the Caatinga indicate an uneven distribution during phenophases, featuring a seasonal vegetation [64,65]. Most tree species flower at the end of the dry season and beginning of the rainy season, while the shrubby and herbaceous formations flower in the rainy season, with the production of flowers soon after the start of the rainy season, and of fruit at the beginning and at the end of the rainy season [63,66–68].

This seasonal characteristic of the vegetation influenced by hydrocycles was the component that most influenced the differences in richness and composition of birds found in this study. A prominent leaf shedding occurs in the vegetation of the region in the dry season with loss of plant biomass and consequently a reduction in the complexity of vegetation structure. This event is directly associated with the smaller bird species richness recorded in the dry season. In the dry/rainy transition season we found a gradual increase in vegetation complexity and species richness, which peaks in the rainy season, when the vegetation also reaches its maximum biomass and increases complexity in regards to vegetation structure. We observed a reduction in vegetation complexity and consequently in species richness when the rainy/dry transition season begins. In this sense, we see that the environmental heterogeneity of Caatinga follows the hydrocycles, where the environment becomes more heterogeneous with increasing precipitation, confirming a pattern well known for birds, where is the richness and species composition of this group of organisms is strongly related with the complexity of vegetation structure [69–71].

Several investigations have shown that seasonal precipitation patterns affect the periodicity of food resources, influencing bird communities [72–74] and the distribution of some organisms [75,76]. Therefore, precipitation can affect bird populations directly through the survival of offspring [77,78], indirectly changing the abundance or availability of invertebrates [28], flowering and fruiting of plant species, and through their impact on the vegetation structure [79]. Illán [80] studied birds of western North America, and highlighted that precipitation was one of the main determinants in the distribution patterns of land birds. Sousa et al. [81] studied birds in the Catimbau National park (Caatinga area) and found strong difference between the richness of birds between the dry and the rainy seasons.

The richness of bird species was explained by local environmental factors, while the composition of bird species was explained by environmental and spatial factors, whereas environmental factors were more important in explaining this variation. Many studies have also found joint relationship between environmental and spatial factors [82–84]. The prevalence of the influence of environmental factors suggests that the mechanisms predicted by the niche theory have a more important role than those predicted by neutral theory in explaining the local structure of bird communities.

However, in addition to the deterministic characteristics and biotic interactions, stochastic processes are also important factors for the distribution of species [85,86]. The neutral theory is the main theoretical support to discuss spatial processes which considers functionally redundant species, attributing their distribution only to spatial processes (stochastic) [12]. However, like our results, other studies that investigated the distribution of birds reported only partial explanations related to spatial processes [9]. Overall, this is why a unified theory that can alone explain the distribution of species will hardly exist [87]. In general, considering the effect of space is very important in community ecology studies [88]. In groups like of birds, where some species have considerable dispersal and movement ability in the landscape, this approach is especially important to prevent type 1 error, because only models based on niche characteristics can be seriously biased due to spatial autocorrelation [89], even when the spatial component of the explanation is low, as in our study. Even considering spatial and environmental aspects together, our models for both richness and composition had high residues. A possible explanation for these residues may be interactions between species such as: predation [90,4], agonistic interactions, intraspecific interaction and competition for food [4,7], which along with spatial and environmental aspects influence the distribution of communities in the landscape [86].

It is important to consider both temporal and spatial variation patterns to investigate diversity patterns [91,92]. Few papers have empirically discussed the mechanisms behind biotic/abiotic relationships considering time and space in the Caatinga. However, several theoretical works have simulated these relations and discussed the importance of the use of temporal and spatial information to understand diversity patterns of communities and/or populations [93,94]. Thus, we believe that our results are a guideline for discussions on bird diversity patterns in the Caatinga, since we investigated the environmental conditions and spatial processes of the communities by considering these relationships throughout the year (hydrocycle).

In general, we find that seasonality in Caatinga affects vegetation structure, influencing the community structure and richness of bird species for each hydrocycle. Our results support other studies carried out in the Caatinga biome, which show that variables linked to climate can be decisive in determining the occurrence and abundance of organisms in xeric regions [16,27,28]. More specifically, our results suggest that vegetation cover and microclimatic temperature affect species richness and composition, while canopy complexity, understory complexity and precipitation affect the composition of bird species.

Currently the predictions on climate change have highlighted frequent dry events and also the increase in global average temperature by 2050 [95]. This would lead to changes in the behavior of some species and to increased desertification in some regions, including the Caatinga region, where global warming induces reduced rainfall by expanding dry climate regions [96,97]. We reasonably conclude, in light of these results, that the climate changes (and thus environmental changes) expected for the future, will lead to significant global changes in species composition at all scales. Our perception that there is more than one factor affecting the species-environment relationship is an important step to understanding such factors, which is important in predicting the effects of global change on biodiversity. With our results we see that the general patterns of climate and vegetation structure directly and indirectly affect the bird species, which are closely linked to habitat quality and landscape conservation status. The high deforestation rates, low level of representativeness of the Caatinga in protected areas and climate change negatively affect the bird communities of the Caatinga. Therefore, this framework shows how climatic factors affect the survival of organisms and thus should be the starting point for any nature conservation initiative to be developed in xeric environments.

## Supporting information

S1 Table Abundance per species in each hydrological season. Raw data of abundance per species per hydrological season in dry, rainy, dry/rainy and rainy/dry transitions collected in the 18 sampling areas.
(PDF)
