# Supplementary material for: The role of climate and environmental variables in structuring bird assemblages in the Seasonally Dry Tropical Forests (SDTFs)
Source: PLoS One. 2017 Apr 25;12(4):e0176066. doi: 10.1371/journal.pone.0176066 (PMC5404791; doi:10.1371/journal.pone.0176066)
Supplement: S1 Table — Raw data of abundance per species per hydrological season in dry, rainy, dry/rainy and rainy/dry transitions collected in the 18 sampling areas. (PDF) [file pone.0176066.s001.pdf]

S1 Table. Abundance per species in each hydrological season.

hydrocycle	sp1	sp2	sp3	sp4	sp5	sp6	sp7	sp8	sp9	sp10	sp11	sp12
dry	0	0	0	0	0	0	0	0	0	0	0	0
rainy	0	0	0	0	1	0	0	0	0	0	0	0
rainy/dry transition	1	0	0	6	0	0	0	0	0	0	0	0
dry/rainy transition	0	0	0	4	1	0	0	0	0	0	0	0
dry	0	0	0	0	1	0	0	0	0	0	0	0
rainy	0	0	8	0	0	0	0	0	0	0	0	0
rainy/dry transition	0	0	1	0	0	0	0	0	0	0	0	0
dry/rainy transition	0	0	0	0	2	0	0	0	0	0	0	0
dry	0	0	0	0	0	0	0	0	0	0	0	0
rainy	0	0	8	0	0	0	0	0	0	0	0	0
rainy/dry transition	1	0	0	0	0	0	0	0	0	0	0	0
dry/rainy transition	0	0	4	0	1	0	0	2	0	0	0	0
dry	0	0	0	0	0	0	0	0	0	0	0	0
rainy	0	0	0	0	0	0	3	2	0	0	0	0
rainy/dry transition	0	0	0	0	0	0	0	0	0	0	0	0
dry/rainy transition	0	0	0	0	0	0	0	0	0	0	0	0
dry	0	0	0	0	0	0	0	0	0	0	0	0
rainy	0	0	5	0	0	0	0	0	0	0	0	0
rainy/dry transition	0	0	0	0	0	0	0	0	0	0	0	0
dry	0	0	0	0	0	0	0	0	0	0	0	0
rainy	1	0	0	0	0	1	0	0	0	0	0	0
rainy/dry transition	0	0	0	0	0	0	0	0	0	0	0	0
dry	0	0	5	0	0	0	0	0	0	0	0	1
rainy	0	0	6	0	3	0	0	0	0	0	0	0
rainy/dry transition	0	0	0	0	0	0	0	0	0	0	0	0
dry/rainy transition	0	0	6	0	0	0	0	0	0	0	0	0
dry	0	0	14	0	1	0	0	0	0	0	0	0
rainy	0	0	4	3	0	0	0	0	0	0	0	0
rainy/dry transition	0	0	2	0	0	0	0	0	0	0	0	0
dry/rainy transition	0	0	3	0	1	0	0	0	0	0	0	0
dry	0	0	21	0	0	0	0	0	0	1	0	0
rainy	0	0	7	0	1	0	0	0	0	0	0	0
rainy/dry transition	1	0	3	0	0	0	0	0	0	1	0	0
dry/rainy transition	1	0	7	0	0	0	0	0	0	0	0	0
dry	0	0	7	0	0	0	0	0	0	0	0	0
rainy	0	0	2	0	0	0	0	2	0	0	0	0
rainy/dry transition	0	0	4	0	0	0	0	2	0	0	1	0
dry/rainy transition	0	0	0	7	0	0	0	2	0	0	0	0
dry	0	0	0	0	2	0	0	0	0	0	0	0
rainy	0	0	1	0	0	0	0	0	0	0	0	0
rainy/dry transition	0	1	0	0	0	0	0	0	0	0	0	0
dry/rainy transition	0	5	0	4	0	2	2	0	0	0	0	0
dry	0	0	0	0	0	0	0	0	0	0	0	0
rainy	0	1	0	0	0	0	0	0	0	0	0	0
rainy/dry transition	0	0	0	0	0	0	0	0	0	0	0	0
dry	0	0	5	0	15	0	0	0	0	0	0	0
rainy	0	0	0	20	0	0	0	1	0	0	0	0
rainy/dry transition	0	0	0	8	1	0	0	0	0	0	0	0
dry/rainy transition	0	0	0	9	0	0	0	0	0	0	0	0
dry	0	2	0	6	4	0	0	0	0	1	0	0
rainy	0	0	0	2	2	0	0	0	0	0	0	0

rainy/dry transition	0	0	0	0	1	0	0	0	2	0	0	0
dry/rainy transition	0	1	0	2	0	0	0	0	0	0	0	0
dry	0	0	0	4	0	0	1	2	0	0	0	0
rainy	0	0	0	6	0	0	0	0	0	0	0	0
rainy/dry transition	0	0	0	6	0	0	0	0	0	0	0	0
dry/rainy transition	1	1	0	13	0	0	0	0	0	0	0	0
dry	0	0	2	0	0	0	0	0	0	0	0	1
rainy	0	0	6	0	0	0	0	0	0	0	0	0
rainy/dry transition	0	0	1	0	0	0	0	0	0	0	0	1
dry/rainy transition	0	0	1	0	1	0	0	0	0	0	0	0
dry	0	0	0	0	0	0	0	0	0	0	0	0
rainy	0	0	0	4	0	0	0	2	0	0	0	0
rainy/dry transition	0	2	1	0	0	0	0	0	3	0	0	0
dry/rainy transition	0	0	0	0	0	0	2	0	0	0	0	0
dry	0	0	2	0	0	0	0	0	0	0	0	0
rainy	0	0	1	0	1	0	0	0	0	0	0	0
rainy/dry transition	0	0	0	0	0	0	0	0	0	0	0	0
dry/rainy transition	0	1	0	0	2	0	0	0	0	0	0	0

sp13	sp14	sp15	sp16	sp17	sp18	sp19	sp20	sp21	sp22	sp23	sp24	sp25	sp26
0	0	0	5	0	0	0	0	1	0	1	0	0	0
0	0	10	11	5	0	0	0	0	0	0	0	0	0
0	0	0	24	8	0	0	1	0	0	0	0	0	0
0	0	6	5	0	0	0	0	0	0	0	0	0	0
0	0	1	2	3	0	0	0	0	0	1	0	0	0
0	0	2	11	12	0	0	0	0	0	2	0	0	0
0	3	2	2	7	0	0	0	4	0	2	0	0	0
0	0	5	2	2	0	0	1	0	0	1	0	1	0
0	0	0	18	6	0	0	0	0	0	0	0	0	0
0	0	2	11	12	0	0	0	0	0	2	0	0	0
0	0	1	10	16	0	0	0	1	0	0	0	1	0
0	1	4	1	1	0	0	0	0	0	1	0	0	0
0	0	1	8	0	0	0	0	0	0	0	0	0	0
0	0	11	26	3	0	0	0	0	0	2	0	0	2
0	0	6	13	8	0	0	0	0	0	0	0	0	0
0	0	7	2	0	0	0	5	0	0	3	0	0	0
0	0	1	2	2	0	0	0	0	0	0	0	0	0
0	1	2	2	5	0	0	0	0	0	0	0	1	0
0	0	2	1	2	0	0	0	0	0	0	0	0	0
0	0	0	4	1	0	0	0	1	0	0	0	0	0
0	0	5	17	11	0	0	0	0	0	5	0	0	0
0	2	2	9	9	0	0	3	0	0	1	0	0	0
0	0	0	0	0	0	0	0	0	0	0	1	0	0
0	0	2	0	3	0	1	1	0	0	1	4	9	0
0	0	1	0	5	0	2	1	2	0	0	0	3	0
0	2	3	0	1	0	0	0	0	0	0	1	0	0
0	0	0	0	0	0	0	0	0	0	0	0	0	0
0	0	4	0	0	0	1	0	0	0	0	2	0	0
0	0	0	0	2	0	2	0	0	0	0	0	2	0
0	0	6	0	0	2	0	3	0	0	0	0	0	0
0	0	0	3	0	2	0	0	0	0	1	1	0	0
0	0	6	2	0	0	3	0	0	0	2	0	0	0
0	5	7	1	2	0	3	2	1	1	2	0	4	0
0	0	4	1	0	0	7	0	0	0	6	1	2	0
0	0	1	10	4	0	0	0	0	0	0	0	0	0
0	0	1	8	4	0	0	0	0	0	1	0	0	2
0	0	3	5	1	1	0	0	1	0	1	0	0	0
0	0	4	0	0	0	0	1	0	0	0	1	1	0
0	0	0	1	2	0	0	0	0	0	3	1	0	0
0	4	0	6	0	0	1	0	0	0	1	0	1	2
0	0	2	7	5	0	0	1	1	0	2	3	1	1
0	0	4	0	0	0	0	1	0	0	4	2	2	0
0	0	0	0	2	0	0	0	0	0	1	0	0	0
0	1	1	1	16	0	0	0	0	0	2	1	1	2
0	0	2	2	11	0	0	0	1	0	1	0	0	0
1	1	0	0	0	0	0	0	0	0	0	15	0	0
0	0	2	0	0	0	4	0	0	0	1	0	0	2
0	0	1	1	0	0	4	0	0	0	2	9	0	0
0	0	3	0	0	0	1	0	2	0	1	1	0	0
0	4	0	1	0	0	0	0	2	0	0	9	0	0
0	1	3	0	2	0	0	0	1	0	0	0	0	0

0	0	2	2	6	0	0	0	0	0	0	11	8	0
0	1	6	0	0	0	0	2	1	0	0	2	4	0
0	0	0	0	0	0	0	0	0	0	0	0	0	0
0	0	0	0	0	0	0	0	0	0	0	1	0	0
0	0	1	2	0	0	5	0	2	0	0	0	1	0
0	0	3	0	0	0	0	1	2	0	0	0	2	0
0	0	0	4	2	0	0	0	0	0	3	0	0	0
0	0	0	5	2	0	0	0	0	0	2	0	0	0
0	2	0	2	1	0	0	1	0	0	0	0	0	0
0	0	0	2	3	0	0	0	0	0	0	0	1	0
0	2	1	1	0	0	0	0	0	0	0	2	0	0
0	0	2	0	3	0	0	0	0	0	1	2	0	0
0	2	1	1	4	0	0	3	0	0	1	1	0	2
0	0	0	0	1	0	0	1	0	0	2	0	3	0
0	0	0	1	0	0	0	0	0	0	0	1	0	0
0	0	1	0	1	0	0	0	0	0	0	0	0	2
0	0	0	1	2	0	0	0	0	0	0	0	0	0
0	0	1	0	2	0	0	0	0	0	0	0	0	0

sp27	sp28	sp29	sp30	sp31	sp32	sp33	sp34	sp35	sp36	sp37	sp38	sp39	sp40
0	0	0	0	0	0	0	0	0	0	0	0	0	0
1	0	0	0	0	0	0	0	7	0	0	0	0	0
0	0	0	0	2	0	1	0	12	2	0	0	0	0
0	0	0	16	0	0	0	2	8	0	0	0	0	0
0	0	0	0	0	0	0	0	0	0	4	0	0	0
0	0	0	0	0	0	0	0	1	0	0	0	0	0
0	0	0	0	0	0	0	0	7	0	0	1	0	0
2	0	0	0	2	0	0	0	1	0	0	0	0	0
0	0	0	0	0	0	0	0	0	0	4	0	0	0
0	0	0	0	0	0	0	0	1	0	0	0	0	0
0	0	0	0	1	0	0	0	0	0	0	0	0	0
0	0	0	0	0	0	0	2	9	0	1	3	0	0
0	0	0	0	0	0	0	0	0	0	0	0	0	0
1	0	0	1	0	2	0	0	0	0	0	0	0	0
0	0	0	0	0	0	0	0	0	0	0	0	0	0
2	0	0	0	0	0	0	0	0	0	0	0	0	0
0	0	0	0	0	0	0	0	7	0	0	0	0	0
0	0	0	0	0	0	0	2	3	0	0	2	0	0
0	0	0	0	0	0	0	0	1	0	0	0	0	0
0	0	0	0	0	0	0	0	2	0	0	0	0	0
0	0	2	0	0	0	0	0	0	0	0	0	0	0
2	0	0	0	0	0	0	0	0	0	0	0	0	0
0	0	0	0	0	0	0	0	0	0	0	0	0	0
0	0	0	1	1	0	0	0	0	0	0	0	0	0
1	0	0	0	0	0	0	0	6	0	0	0	0	0
0	0	0	3	0	0	0	0	6	0	0	0	0	0
0	0	0	0	0	0	0	0	3	0	3	0	0	0
0	0	0	0	0	0	0	1	14	0	0	0	0	0
0	0	0	0	0	0	0	0	4	1	0	0	0	0
0	0	0	2	0	0	0	0	10	0	0	0	0	0
0	0	0	0	0	0	1	0	8	0	0	0	0	0
1	0	0	0	0	0	0	0	5	3	0	2	0	0
4	0	0	0	0	0	0	1	25	0	0	0	0	2
1	0	0	0	0	0	0	0	6	1	0	5	0	0
0	0	0	0	0	0	0	0	0	0	9	0	0	0
1	0	0	0	0	0	0	1	1	0	0	0	0	0
1	0	0	0	0	0	0	0	1	0	0	0	0	0
0	0	0	0	0	0	0	0	3	0	0	0	0	0
0	0	0	0	0	0	0	0	0	0	0	0	0	0
2	0	0	0	0	0	0	0	2	0	1	8	0	0
2	0	0	0	0	0	0	0	0	0	2	21	0	0
0	0	0	3	3	0	0	1	2	0	4	5	0	0
0	0	0	0	1	0	0	0	0	0	0	0	0	0
0	0	1	1	0	0	0	1	0	0	0	2	0	0
0	0	0	0	0	0	0	0	0	0	0	0	0	0
0	0	0	0	1	0	0	0	0	0	0	0	0	0
0	0	0	0	0	0	0	0	9	2	0	0	0	0
1	0	0	0	0	0	3	0	24	0	3	4	0	0
0	0	0	0	0	0	0	2	16	0	4	0	0	0
0	0	0	0	0	0	1	0	0	0	5	0	0	0
1	0	0	0	2	0	0	1	7	0	0	0	0	0

0	0	0	0	0	0	0	0	3	0	0	7	0	0
0	0	0	2	0	0	0	0	19	0	4	0	0	0
0	0	0	0	0	0	0	0	0	0	0	0	0	0
0	0	0	0	0	0	0	0	4	0	0	0	0	0
2	0	0	0	0	0	1	0	27	3	5	8	0	0
0	0	0	0	0	0	0	4	8	0	0	1	0	0
0	0	0	0	0	0	0	0	0	0	0	0	0	0
1	0	0	0	0	0	1	0	0	0	0	5	0	0
0	0	0	0	0	0	0	0	0	0	0	0	0	0
0	2	0	0	0	0	0	0	0	0	0	3	0	0
0	0	0	0	0	0	0	0	0	4	0	0	1	0
4	0	0	0	2	0	1	0	4	0	0	5	1	0
0	0	0	0	0	0	0	0	3	0	0	3	6	0
3	0	0	0	3	0	0	0	5	0	0	7	0	0
0	0	0	0	0	0	0	0	0	0	0	0	1	0
0	0	0	0	0	0	0	0	1	0	1	0	0	0
1	0	0	0	0	0	0	0	0	0	0	5	3	0
0	0	0	0	1	0	0	0	0	0	1	1	0	0

sp41	sp42	sp43	sp44	sp45	sp46	sp47	sp48	sp49	sp50	sp51	sp52	sp53	sp54
0	0	0	1	3	0	0	3	3	1	0	0	0	0
0	0	0	3	0	0	9	5	0	1	0	0	6	0
0	0	0	0	0	1	2	6	0	0	0	0	0	0
0	9	0	0	0	0	7	1	0	0	0	0	0	0
0	0	0	1	3	0	0	0	0	0	11	0	0	0
0	1	3	2	0	0	19	0	0	0	0	0	0	0
0	0	0	0	14	0	1	0	2	0	0	0	0	0
0	9	4	0	0	0	7	0	0	0	0	0	0	0
0	0	1	0	1	0	0	0	0	0	0	0	0	0
0	1	3	2	0	0	19	0	0	0	0	0	0	0
0	0	0	0	0	0	4	0	0	0	0	0	0	0
0	13	5	0	0	0	10	0	0	0	0	0	0	0
0	1	0	0	4	0	0	4	0	0	0	0	0	0
0	0	0	0	6	0	10	10	0	2	0	1	0	0
0	0	0	0	2	0	8	2	0	0	0	0	0	0
0	9	0	0	1	0	13	1	0	1	0	0	0	1
0	0	0	0	8	0	0	0	0	1	0	0	0	0
0	0	1	6	5	0	28	0	0	0	0	0	0	1
0	0	0	0	1	3	3	0	0	0	0	0	0	0
0	0	0	0	8	0	0	1	0	2	0	0	0	0
0	4	0	0	15	0	11	3	0	9	0	0	0	0
0	0	1	0	3	0	1	1	0	0	0	0	0	0
0	0	1	0	12	0	0	0	0	0	0	0	0	0
0	0	0	4	2	1	6	0	2	0	0	0	0	1
0	0	0	3	4	0	0	1	0	0	0	0	0	0
0	0	1	0	0	1	2	0	0	0	0	0	0	0
0	0	0	0	5	0	0	0	0	2	1	0	0	0
2	0	3	2	3	0	7	0	0	0	0	0	4	0
0	0	0	0	3	1	1	1	0	0	0	0	0	0
0	0	0	0	2	1	11	0	0	0	0	0	0	0
0	0	0	0	19	7	0	0	0	0	0	0	0	0
0	0	0	2	1	0	0	3	5	0	0	0	0	2
0	0	0	0	8	3	1	0	0	0	0	0	0	1
0	1	2	0	9	2	2	0	0	0	1	0	0	0
0	0	0	0	1	0	0	0	0	0	0	0	0	0
0	0	0	2	0	0	17	0	0	0	0	0	0	0
0	0	0	0	0	0	4	0	0	0	0	0	0	0
0	2	0	0	6	0	10	2	0	0	0	0	0	0
0	0	0	0	14	2	0	0	0	0	0	0	0	0
0	0	0	1	11	0	1	0	0	0	0	0	0	1
0	0	3	0	12	0	5	2	0	0	0	0	0	0
0	0	1	4	1	0	5	0	0	0	0	0	0	2
0	0	0	0	7	1	0	0	0	0	0	0	0	0
0	3	1	3	3	0	15	0	0	0	1	0	1	0
0	0	1	0	3	0	2	0	0	0	0	0	0	0
0	0	0	1	6	0	0	0	0	2	3	0	0	0
0	0	2	0	14	0	0	1	0	2	0	0	5	0
0	0	0	0	11	0	0	3	0	2	0	0	0	0
0	1	0	0	7	0	2	1	0	0	0	0	0	0
0	0	0	0	10	0	0	2	0	1	0	0	0	0
0	0	0	0	7	1	6	1	1	0	0	0	0	2

0	0	1	0	1	2	4	2	0	2	0	0	0	0
0	0	0	0	7	0	6	0	0	1	0	0	0	4
0	0	0	0	5	3	0	1	0	0	0	0	0	0
0	0	0	0	0	0	3	1	0	1	0	2	0	0
0	0	0	0	5	0	1	5	0	4	0	0	0	0
0	0	0	3	2	0	1	0	0	3	0	0	0	1
0	0	0	0	2	0	0	0	0	0	0	0	0	0
0	0	0	1	3	1	8	0	0	0	0	0	0	0
0	0	0	0	0	0	3	1	0	0	0	0	0	0
0	13	5	0	0	0	7	0	0	0	0	0	0	0
0	0	0	0	5	0	0	2	0	2	0	0	0	0
0	0	0	0	17	0	1	8	0	1	0	0	0	0
0	0	0	0	4	0	0	14	0	0	0	0	0	0
0	2	2	0	6	0	4	3	0	0	0	0	0	1
0	0	0	1	15	0	2	3	0	0	0	0	0	0
0	0	0	0	1	0	6	2	0	0	0	0	0	0
0	0	0	0	2	0	1	2	1	0	0	0	0	0
0	5	2	1	0	0	0	0	0	0	0	0	0	0

sp55	sp56	sp57	sp58	sp59	sp60	sp61	sp62	sp63	sp64	sp65	sp66	sp67	sp68
0	0	0	0	0	0	0	24	0	0	0	0	0	0
0	0	13	0	0	0	10	4	0	0	0	0	0	0
0	0	0	0	0	0	0	12	0	0	0	0	0	0
0	2	14	0	0	0	0	14	0	0	0	0	0	0
0	3	13	0	0	0	0	23	0	0	0	0	0	0
0	0	3	1	0	0	0	15	0	0	0	0	0	0
0	0	2	1	0	0	0	10	0	0	0	0	0	0
0	2	7	6	0	0	0	6	0	0	0	0	0	0
0	0	0	0	0	0	0	26	0	0	0	0	0	0
0	0	3	1	0	0	0	15	0	0	0	0	0	0
0	0	1	0	0	0	0	2	0	0	0	0	0	0
0	1	4	1	0	0	0	8	0	0	0	0	0	0
0	1	0	0	0	0	0	13	0	0	0	0	0	0
0	0	7	0	0	0	2	4	0	0	0	0	0	0
0	0	0	0	0	0	0	2	0	0	0	0	0	0
0	1	6	0	0	0	0	8	0	0	0	0	0	0
0	2	7	0	0	0	0	7	0	0	0	0	0	0
0	2	1	0	0	0	0	7	0	0	0	0	0	0
0	1	0	0	0	0	0	3	0	0	0	0	0	0
0	0	4	0	0	0	0	8	0	0	0	0	0	0
0	0	4	0	0	0	0	10	0	0	0	0	0	0
0	0	0	0	0	0	1	5	0	0	0	0	0	0
0	4	2	0	0	0	0	9	0	0	0	0	0	0
1	0	5	9	0	0	0	13	0	5	0	0	0	0
0	2	2	0	0	0	0	6	0	0	0	0	0	0
0	0	5	12	0	0	0	11	3	0	0	0	0	0
0	1	19	0	0	0	0	9	0	0	1	0	0	0
0	0	7	0	0	0	2	10	0	0	0	0	0	0
0	0	0	0	0	0	0	2	0	0	0	0	0	0
0	0	0	6	0	0	0	5	0	0	0	0	0	0
0	15	30	0	0	0	0	12	0	0	0	0	0	0
0	7	6	2	0	0	0	7	0	5	1	0	0	0
0	1	2	0	0	1	0	4	0	6	1	0	1	0
0	15	7	0	0	0	0	2	0	0	0	0	0	0
0	0	0	0	0	0	0	18	0	0	0	0	0	1
0	1	0	0	0	0	0	7	0	0	0	0	0	0
0	2	8	0	0	0	0	2	0	0	0	0	0	0
0	2	44	0	0	0	0	2	0	0	0	0	0	0
0	2	21	0	0	0	0	13	0	22	0	0	0	2
0	1	3	0	0	0	0	6	0	23	0	0	0	0
0	0	2	0	0	0	0	6	0	15	1	0	0	0
0	0	14	1	0	0	0	2	1	10	0	0	0	0
0	3	0	0	0	0	0	9	0	1	0	0	0	0
0	0	0	0	0	0	0	17	0	0	0	0	0	0
0	0	0	0	0	0	0	3	0	0	0	0	0	0
0	2	0	0	0	0	0	3	0	0	1	1	0	1
0	1	0	0	1	2	0	0	0	0	0	0	0	1
0	0	0	0	0	2	0	2	0	0	0	0	0	0
0	0	0	0	0	0	0	0	0	0	0	0	0	2
0	1	4	0	0	0	0	5	0	0	0	0	0	1
0	1	4	0	0	0	0	7	0	0	0	0	0	0

0	0	0	0	0	0	0	7	0	0	0	0	0	0
0	1	6	0	0	0	0	2	0	0	0	0	0	0
0	0	0	0	0	0	0	0	0	0	0	0	0	0
0	0	0	0	0	0	0	0	0	0	0	0	0	0
0	0	6	0	0	0	0	0	0	0	0	0	0	0
0	0	2	0	0	0	0	2	0	0	0	0	0	0
0	1	0	0	0	0	0	21	0	0	0	0	0	0
0	2	0	0	0	0	0	1	0	0	0	0	0	0
0	0	0	0	0	0	0	4	0	0	0	0	0	0
0	0	0	0	0	0	0	8	0	0	2	0	0	0
0	0	12	0	0	0	0	4	0	0	0	0	0	0
0	0	13	0	0	0	0	6	0	0	0	0	0	0
0	2	0	0	0	0	0	2	0	0	2	0	0	0
0	3	0	0	0	0	0	0	2	0	0	0	0	0
0	0	35	0	0	0	0	8	0	1	0	0	0	0
0	4	3	0	0	0	0	5	0	0	0	0	0	0
0	0	1	0	0	0	0	1	0	0	0	0	0	0
0	0	0	0	0	0	0	2	0	0	0	0	0	0

sp69	sp70	sp71	sp72	sp73	sp74	sp75	sp76	sp77	sp78	sp79	sp80	sp81	sp82
0	0	0	0	0	0	14	0	2	3	0	2	0	0
0	0	0	0	0	0	18	0	1	14	0	0	0	0
0	0	0	0	0	0	9	0	0	18	0	0	0	0
0	0	0	0	0	0	6	2	0	7	0	0	0	0
0	0	0	0	0	0	0	0	0	6	0	0	0	0
0	0	0	0	0	0	9	0	0	10	0	0	0	0
0	2	0	0	0	0	4	1	0	2	0	0	0	0
0	0	0	0	0	0	12	0	0	5	0	0	0	0
0	0	0	0	0	0	10	0	0	10	0	0	0	0
0	0	0	0	0	0	9	0	0	10	0	0	0	0
1	0	0	0	0	0	6	0	0	8	0	0	0	0
0	0	0	0	0	0	5	0	0	2	0	0	0	0
0	1	0	0	0	0	16	0	2	0	0	0	1	0
0	3	0	2	0	0	5	0	0	12	0	0	0	0
0	0	0	0	0	0	6	0	0	4	0	0	0	0
0	1	0	0	0	0	7	0	0	2	0	0	0	0
0	0	4	0	0	0	11	0	0	1	0	1	0	0
0	0	0	0	0	0	7	1	0	7	0	0	0	0
0	0	0	0	0	0	4	1	0	0	0	0	0	0
0	0	0	0	0	0	8	0	0	0	0	0	0	0
0	1	0	0	0	0	7	0	0	11	0	0	0	0
0	2	0	0	0	0	5	0	0	9	1	0	2	0
0	0	0	0	0	0	2	0	0	1	0	0	0	0
0	0	0	0	0	0	3	0	0	6	0	0	0	0
0	1	0	0	0	0	8	0	0	3	0	0	0	0
0	0	3	0	0	0	5	0	0	2	0	0	0	0
0	1	0	0	0	0	9	1	0	2	0	1	0	0
0	0	4	0	0	0	14	0	0	2	0	0	1	0
0	0	0	0	0	0	9	0	0	2	0	0	0	0
0	0	0	0	0	0	11	0	0	0	0	0	0	0
0	0	0	0	0	0	4	0	2	3	0	2	0	0
0	6	0	0	0	0	2	2	0	2	0	0	0	0
0	1	0	0	0	0	4	0	0	6	0	0	0	0
1	2	0	0	0	1	6	0	0	6	0	1	0	0
0	0	0	0	0	0	1	0	0	4	0	0	1	3
0	0	0	0	0	0	3	0	0	0	0	0	3	0
0	0	0	0	0	0	0	0	0	2	0	0	0	0
0	0	0	0	0	0	1	0	0	1	0	0	0	0
0	1	5	0	0	0	2	0	0	3	0	1	0	0
0	2	0	0	0	0	3	1	0	4	0	0	0	0
0	2	0	0	0	0	2	1	0	2	0	0	2	0
0	1	0	0	0	6	3	0	0	0	0	0	0	0
0	0	0	0	0	0	2	0	0	7	0	0	0	0
0	1	0	0	0	0	1	0	0	5	0	0	0	0
0	3	0	0	0	0	0	0	0	5	0	0	0	0
0	0	0	0	0	0	4	0	2	1	0	6	1	0
0	1	9	0	0	0	2	0	2	0	0	12	0	0
0	3	1	0	0	6	6	0	2	0	0	13	0	0
0	1	0	0	1	8	3	0	0	0	0	0	0	0
0	2	0	0	2	0	5	1	0	0	0	16	0	2
0	2	0	0	0	0	5	0	0	4	0	0	0	0

0	2	0	0	0	6	10	0	1	0	0	3	0	0
0	0	0	0	0	2	8	0	0	0	0	0	0	0
0	2	0	0	0	0	4	0	3	0	0	4	0	0
0	2	0	0	0	0	2	0	0	0	0	8	6	2
0	1	0	0	0	10	8	0	2	0	0	20	0	0
0	1	2	0	0	6	6	1	0	0	0	2	0	0
0	1	0	0	0	0	1	0	1	6	0	0	0	0
0	0	0	0	0	0	6	0	0	4	0	0	0	0
0	0	0	0	0	0	4	0	0	0	0	0	0	0
0	0	0	0	0	0	12	0	0	1	0	0	0	0
0	2	0	0	0	0	2	1	4	0	0	8	1	0
0	2	0	0	0	7	1	1	1	3	0	3	0	0
2	1	0	0	0	8	4	0	4	1	0	2	0	0
0	1	0	0	0	4	0	0	0	0	0	3	0	0
0	1	0	0	0	0	7	0	4	1	0	9	2	0
0	0	0	0	0	0	3	0	0	0	0	5	0	0
0	0	0	0	0	0	3	0	1	0	0	1	0	0
0	0	4	0	0	5	3	0	0	0	0	1	0	0

sp83	sp84	sp85	sp86	sp87	sp88	sp89	sp90	sp91	sp92	sp93	sp94	sp95	sp96
0	1	0	0	0	0	0	0	0	0	0	0	0	1
0	31	1	0	0	14	0	6	0	0	6	0	0	1
0	47	7	0	0	46	0	1	0	0	3	0	0	1
0	14	7	0	0	16	0	2	0	0	6	3	0	0
0	1	0	0	0	0	0	4	0	0	0	0	0	0
3	20	0	0	1	6	1	1	0	0	2	3	0	0
2	9	1	0	0	3	0	5	0	0	0	6	0	0
0	18	10	0	0	2	0	0	0	0	0	3	0	0
0	8	1	0	0	0	0	8	0	2	0	0	0	0
3	20	0	0	1	6	1	1	0	0	2	3	0	0
0	25	1	0	2	0	0	1	0	0	0	1	0	1
0	5	2	0	0	27	0	6	0	0	0	6	0	2
0	4	0	0	0	0	0	1	0	0	0	0	0	1
5	10	0	0	2	5	1	6	0	0	1	0	0	5
2	4	1	0	0	11	0	3	0	0	0	0	1	1
0	8	1	0	0	12	0	2	0	0	4	0	0	0
0	2	0	2	0	5	0	2	0	0	0	0	0	1
0	18	0	0	1	4	0	3	0	0	0	5	0	0
0	2	1	0	0	2	0	0	0	0	0	0	1	0
0	2	0	0	0	0	0	2	0	0	0	0	0	0
0	23	0	0	0	14	2	0	0	1	3	0	0	1
3	18	9	0	0	4	0	1	0	0	0	0	0	2
0	3	0	0	0	2	0	0	0	1	0	0	0	0
0	7	1	0	0	8	0	1	0	0	0	2	0	0
0	7	1	0	0	2	0	6	0	0	4	0	0	1
0	0	3	0	0	12	0	2	0	0	0	3	0	0
0	0	0	1	0	0	0	5	0	1	0	0	0	0
2	0	0	0	0	2	0	9	0	0	5	0	0	0
0	0	0	0	0	6	0	1	0	0	0	3	0	0
0	1	4	0	0	11	0	6	0	0	0	4	0	0
0	3	0	0	0	0	0	9	0	0	0	0	0	2
0	6	0	0	1	6	0	13	0	0	0	12	1	0
0	6	1	0	2	17	0	7	0	0	0	3	0	0
0	2	0	0	0	24	0	7	0	0	1	3	2	0
0	8	0	0	0	0	0	5	0	0	0	0	2	1
0	11	0	0	0	6	0	5	0	0	0	0	1	0
0	8	0	0	1	3	0	2	0	0	0	0	0	0
0	6	0	0	0	4	0	6	0	0	3	0	0	0
0	2	0	0	0	1	0	3	0	0	0	0	0	1
3	11	0	0	1	27	0	6	0	0	1	0	0	0
0	9	0	0	2	0	0	10	0	0	0	2	1	0
0	1	0	0	2	10	0	10	0	0	1	0	2	0
0	0	2	0	0	4	0	0	0	0	0	0	0	0
0	1	0	0	0	30	0	0	0	0	0	0	2	0
0	3	0	0	0	8	0	0	0	0	0	1	2	0
0	0	1	0	0	0	0	1	0	0	0	0	0	1
1	0	0	3	0	4	0	4	0	0	21	0	4	0
0	0	0	1	0	4	0	7	0	0	32	0	2	1
0	0	0	2	0	3	0	2	0	0	6	3	4	0
0	0	0	0	1	0	0	1	0	0	0	0	0	0
1	0	0	2	0	6	0	3	0	0	12	1	6	1

0	1	0	0	1	12	0	9	0	0	9	2	7	1
0	0	0	0	0	7	0	6	0	0	7	2	1	0
0	0	0	0	0	0	0	0	0	0	0	0	0	1
9	0	0	0	0	2	0	2	0	0	0	0	0	2
0	0	0	0	0	2	0	5	0	0	15	0	1	0
0	0	0	0	0	5	0	4	0	0	6	0	1	0
0	7	0	0	0	0	0	13	0	0	0	0	0	0
2	13	0	0	0	3	0	8	0	0	1	0	0	0
0	7	0	0	0	7	0	0	0	0	0	0	1	0
0	4	2	0	0	18	0	2	0	0	0	3	0	0
1	0	2	0	0	0	0	3	1	0	0	0	1	1
0	3	0	0	2	0	0	8	2	0	4	0	7	2
0	17	0	0	0	4	0	2	0	0	2	1	3	1
0	0	0	0	0	0	0	1	0	1	0	11	2	1
0	1	3	0	0	0	0	7	0	0	0	1	0	0
0	4	0	0	0	0	0	1	0	0	9	2	0	0
0	5	0	0	0	0	0	2	0	1	0	1	3	0
0	2	0	0	0	0	0	6	0	2	2	4	0	0

sp97	sp98	sp99	sp100	sp101	sp102	sp103	sp104	sp105	sp106	sp107	sp108
2	0	0	0	0	0	0	0	0	0	0	0
1	0	2	0	0	0	0	0	15	0	0	8
17	0	2	0	0	0	0	0	40	0	0	0
9	0	3	0	0	0	3	1	8	0	0	9
0	0	0	0	0	0	0	5	5	0	0	0
7	0	0	0	0	0	1	0	8	0	0	13
6	0	0	1	0	0	0	0	12	0	0	0
10	0	0	0	0	0	0	5	5	0	0	7
5	0	0	0	0	0	0	0	5	0	0	0
7	0	0	0	0	0	1	0	8	0	0	13
14	0	0	0	0	0	0	0	19	0	1	0
2	0	0	0	0	0	1	0	2	0	5	7
3	0	5	0	0	0	0	0	0	0	0	0
10	0	2	0	0	0	0	0	42	0	2	1
9	0	2	1	0	0	0	0	34	0	0	0
7	0	2	0	0	0	2	0	13	0	0	5
10	0	3	0	0	0	0	0	1	0	0	0
15	0	3	0	0	0	0	0	10	0	3	2
7	0	3	0	0	0	0	0	4	0	0	0
7	0	2	0	0	0	0	0	0	0	0	0
17	0	5	0	0	0	0	0	48	0	3	22
10	0	7	2	0	0	0	2	28	0	0	0
5	0	0	0	0	0	0	0	0	0	0	0
4	0	1	0	0	3	0	0	6	0	0	3
4	0	2	0	0	0	0	2	4	0	0	2
2	0	0	0	0	0	0	3	3	0	0	7
1	0	1	0	0	0	1	0	0	0	0	0
0	0	3	0	0	0	0	1	12	0	0	3
0	0	4	0	0	0	0	0	7	0	0	0
2	0	3	0	0	0	0	0	5	0	3	8
6	0	1	0	0	0	0	0	0	0	1	0
5	0	3	0	0	0	0	3	10	0	21	9
4	0	0	0	0	0	0	4	8	0	1	0
3	0	1	0	0	0	0	5	11	0	8	7
8	0	5	0	0	0	0	0	11	0	0	0
11	0	1	0	0	0	0	0	8	0	2	7
7	0	4	0	0	0	0	0	10	0	0	0
3	0	1	0	1	0	0	0	4	0	1	2
5	0	1	0	0	0	0	0	2	0	0	0
3	0	0	0	0	0	0	3	4	0	4	11
5	0	6	1	1	0	0	1	3	1	1	8
1	0	2	2	0	0	0	0	4	0	3	8
2	0	1	0	0	0	0	0	7	0	0	0
3	0	2	0	0	0	0	1	13	0	5	3
7	0	7	0	0	0	0	0	8	0	0	0
0	0	1	0	0	0	1	0	0	0	0	0
0	0	3	0	0	0	0	0	8	0	3	18
0	0	3	0	0	0	0	0	17	0	1	6
1	1	2	0	0	0	6	0	5	0	0	10
0	0	3	0	0	0	1	0	0	0	0	0
2	0	3	0	0	0	0	0	18	0	2	25

3	0	3	0	0	0	0	0	17	0	0	1
2	0	1	0	0	0	3	0	1	0	1	6
0	0	3	0	0	0	0	0	0	0	0	0
0	0	2	0	0	0	0	3	0	0	3	6
0	0	3	0	0	0	0	0	10	0	0	4
1	0	0	0	0	0	0	0	6	0	2	9
1	0	2	0	0	0	0	0	8	0	0	0
9	0	0	2	0	0	2	3	3	0	3	9
5	0	2	3	0	0	0	0	9	0	0	0
4	0	2	1	0	0	0	0	3	0	0	16
1	0	3	0	0	0	0	0	2	4	0	0
0	0	5	0	0	0	0	0	10	0	2	29
1	0	6	1	0	0	0	0	11	4	1	12
0	0	2	0	0	0	0	0	1	0	5	3
10	0	5	0	0	0	0	0	1	2	0	0
2	0	4	0	0	0	0	2	5	0	3	5
4	0	6	1	0	0	0	0	8	0	2	2
4	0	1	1	0	0	0	0	2	0	8	8

sp109	sp110	sp111	sp112	sp113	sp114	sp115	sp116	sp117	sp118	sp119
0	0	8	0	0	0	0	1	0	0	0
0	0	34	0	0	0	0	2	0	0	0
0	0	42	0	0	0	0	0	0	0	0
0	0	19	0	0	0	0	5	5	0	0
0	0	7	0	0	0	0	0	0	0	0
0	0	28	0	0	0	0	5	0	0	0
0	0	10	1	0	0	0	2	0	0	0
0	0	2	0	0	0	0	0	1	0	0
0	0	11	0	0	0	0	0	0	0	0
0	0	28	0	0	0	0	5	0	0	0
0	0	12	0	0	0	0	3	0	0	0
0	0	7	7	0	0	0	6	8	0	0
0	0	15	0	0	0	0	0	0	0	0
0	0	12	0	0	0	0	5	1	0	0
0	0	7	0	0	0	0	0	0	0	0
0	0	10	0	0	0	0	4	6	0	0
0	0	13	0	0	0	0	0	0	0	0
0	2	20	0	0	0	0	3	2	0	0
0	1	7	0	0	0	0	0	0	0	0
0	0	9	0	0	0	0	0	0	0	0
0	0	25	0	1	0	1	5	7	0	0
1	1	17	0	0	0	1	0	0	0	0
0	0	4	0	0	0	0	0	0	0	0
0	0	18	3	0	0	0	0	4	0	3
0	1	18	0	0	0	0	0	1	0	0
0	1	12	0	0	0	0	0	0	0	3
0	0	5	0	0	0	0	0	0	0	0
0	1	8	0	0	0	0	9	1	0	1
0	1	2	0	0	0	0	0	0	0	0
0	6	3	1	0	0	0	0	0	0	5
0	0	8	0	0	0	0	7	0	0	0
0	0	4	4	0	0	0	3	9	0	0
0	0	7	1	0	0	0	3	0	0	0
0	3	7	5	0	0	0	0	10	0	0
0	0	19	0	0	0	1	0	0	0	0
0	0	14	1	0	0	0	4	2	0	0
0	1	5	1	0	0	0	0	0	0	2
0	2	8	0	0	0	0	1	4	0	0
0	0	10	0	1	0	0	0	0	0	0
0	6	18	4	0	0	0	9	14	0	0
0	5	7	4	0	2	0	5	5	1	0
0	11	6	6	0	0	0	5	8	0	0
0	0	0	0	0	0	0	0	0	0	0
0	0	0	0	0	0	0	0	2	0	0
1	2	0	0	0	0	0	0	0	0	0
0	0	4	0	0	0	0	1	0	0	0
0	0	0	0	1	0	0	5	1	0	0
0	0	1	8	0	0	0	5	0	0	0
0	2	0	3	0	0	0	0	3	0	0
0	0	0	0	0	0	0	1	0	0	0
0	1	8	5	0	0	0	2	2	0	0

0	2	3	7	0	0	0	5	0	0	0
0	5	5	2	0	0	0	1	7	0	0
0	0	0	0	0	0	0	0	0	0	0
0	0	0	0	0	0	1	2	0	0	0
0	6	0	17	0	0	0	0	0	0	0
0	14	0	5	0	0	0	1	3	0	0
0	0	16	0	0	0	0	1	0	0	0
0	0	20	6	0	0	1	6	8	0	0
0	1	5	0	0	0	0	0	0	0	0
0	6	7	4	2	0	0	0	9	0	0
0	0	5	0	2	3	0	0	0	0	0
0	11	6	9	1	3	0	1	4	2	0
0	4	2	9	3	0	0	6	0	1	0
0	1	3	7	3	0	0	0	3	0	0
0	0	8	0	1	0	0	0	0	0	0
0	4	7	1	0	0	0	1	1	0	0
0	9	3	3	0	1	0	2	0	0	0
0	10	1	3	1	1	0	0	2	0	0

sp120	sp121	sp122	sp123	sp124	sp125	sp126	sp127	sp128	sp129	sp130
0	0	0	0	0	0	0	1	1	0	0
0	0	0	16	0	0	1	6	1	0	0
0	0	0	8	0	0	0	2	0	0	0
0	0	0	3	3	0	1	0	1	0	0
0	0	0	0	0	0	0	0	2	0	0
1	0	0	0	0	0	0	1	3	0	0
0	0	0	1	0	0	0	1	7	0	0
0	0	0	7	0	0	2	1	0	0	0
0	0	0	0	0	0	0	0	4	0	0
1	0	0	0	0	0	0	1	3	0	0
0	0	0	0	0	0	0	0	0	0	0
0	0	0	5	0	0	0	0	3	0	0
0	0	0	0	0	0	0	0	1	0	0
0	0	0	6	0	0	0	0	2	0	0
0	0	0	2	0	0	0	1	1	0	0
0	0	3	9	0	0	0	0	1	0	0
0	0	0	0	0	0	0	0	3	0	0
0	0	0	1	0	0	1	2	0	0	0
0	0	0	0	0	0	0	0	0	0	0
0	0	0	0	0	0	0	0	5	0	0
0	0	2	7	0	0	2	0	3	0	0
0	0	0	2	0	0	0	0	2	0	0
0	0	0	0	0	0	0	0	3	0	0
0	0	0	4	0	0	0	0	4	0	0
0	0	1	3	0	0	0	0	1	0	0
0	0	0	11	0	0	1	1	1	0	0
0	0	0	0	0	2	0	1	1	0	0
0	0	0	3	0	0	0	0	0	0	0
0	0	0	2	0	0	0	0	1	0	0
0	0	0	4	0	0	0	0	2	2	0
0	0	4	0	0	0	0	0	6	0	0
18	1	0	1	0	0	0	0	0	0	0
0	0	0	3	0	0	0	0	2	0	0
0	0	0	3	0	0	1	0	0	0	0
0	0	0	0	0	0	0	0	2	0	0
1	0	0	0	0	0	0	2	0	0	0
0	0	0	1	0	0	0	1	0	0	0
0	0	1	5	0	0	0	0	1	0	0
0	0	0	0	0	0	0	2	1	0	0
0	0	0	0	0	0	2	1	0	6	0
0	0	0	1	0	0	0	4	3	0	0
0	0	0	0	0	0	0	0	0	0	0
0	0	0	0	0	0	0	2	2	0	0
0	0	1	5	0	0	1	1	3	0	0
0	0	0	2	0	0	0	1	0	0	0
0	0	0	0	0	0	0	1	2	0	0
0	0	2	0	1	0	3	0	0	0	0
0	0	0	3	0	0	0	2	0	0	0
0	0	0	5	0	0	1	0	0	0	0
0	0	0	0	0	0	0	0	0	0	0
0	0	0	1	0	0	2	0	2	0	0

0	0	0	0	0	0	0	0	5	0	0
0	0	0	2	0	0	0	0	1	0	0
0	1	5	0	0	0	0	0	2	0	0
0	0	0	0	0	0	1	0	0	0	0
0	0	0	2	0	0	0	1	1	0	0
0	0	2	4	0	0	2	0	0	0	0
0	0	0	0	0	0	0	2	2	0	0
0	0	0	1	0	0	0	1	2	0	0
0	0	0	0	0	0	0	1	1	0	0
0	0	0	5	0	0	0	0	3	0	0
0	0	0	0	0	1	0	1	1	0	0
0	0	0	6	2	0	3	3	1	0	0
0	0	0	1	1	0	0	1	0	0	0
0	0	0	0	0	0	1	1	3	0	0
0	0	0	0	0	0	0	0	1	0	0
1	0	0	0	0	0	0	0	0	1	0
0	0	0	0	0	0	0	0	1	0	2
0	0	2	0	0	0	1	0	0	1	0

sp131	sp132	sp133	sp134	sp135	sp136	sp137	sp138	sp139	sp140	sp141
8	0	0	0	0	0	1	8	0	0	0
6	0	0	0	0	0	0	43	0	0	0
28	0	0	0	0	0	1	32	0	0	0
13	0	0	0	0	0	1	24	0	0	0
10	0	0	2	0	0	0	3	0	0	0
11	0	0	0	0	0	1	35	0	0	0
2	0	0	0	0	0	3	7	0	0	0
5	0	0	0	0	0	2	23	0	0	0
7	0	0	0	0	0	0	8	0	0	0
11	0	0	0	0	0	1	35	0	0	0
9	0	0	0	0	0	1	18	0	0	0
6	0	0	0	0	0	3	9	0	0	0
6	0	0	0	0	0	0	12	0	0	0
17	0	0	0	0	0	2	13	0	0	0
11	0	0	0	0	0	0	15	0	0	0
12	0	0	0	0	0	1	21	0	0	0
6	0	0	0	0	0	0	0	1	0	0
3	0	0	0	0	0	1	11	0	0	0
7	0	0	0	0	0	0	2	0	0	0
12	0	0	0	0	0	3	0	0	0	0
12	0	0	0	0	0	1	15	0	0	0
23	0	0	0	0	0	0	3	0	0	0
5	0	0	0	0	0	0	3	0	0	0
0	0	0	0	0	0	2	12	1	1	0
6	0	0	0	0	1	1	12	0	0	0
3	0	0	0	0	0	2	9	0	0	0
2	0	0	2	0	0	0	0	0	0	0
0	0	0	0	0	0	1	2	0	0	0
4	0	0	0	0	0	0	1	0	0	0
5	0	0	0	0	0	0	0	0	0	0
8	0	0	4	0	0	0	7	0	0	0
0	1	0	0	0	0	1	7	0	0	0
7	0	1	0	0	0	3	2	0	0	0
1	0	0	5	0	0	3	8	0	0	0
9	0	0	0	0	0	0	8	1	0	0
1	0	0	0	0	0	0	14	0	0	0
6	0	0	0	0	0	2	3	0	0	0
2	0	0	0	0	0	0	12	1	0	0
3	0	0	0	0	0	1	3	0	0	0
1	0	8	0	1	0	1	10	0	0	0
2	0	0	0	0	0	5	1	0	0	0
0	0	0	0	0	0	0	3	0	0	0
2	0	0	0	0	0	0	1	0	0	0
0	0	0	0	0	0	3	0	0	0	0
0	0	0	0	0	0	2	1	0	0	0
8	0	0	0	0	0	0	1	0	0	0
0	4	0	0	0	0	1	0	0	0	0
3	0	0	0	0	0	1	0	0	0	0
0	0	0	0	0	0	2	0	0	0	0
12	0	0	0	0	0	1	0	0	0	0
1	2	0	0	0	0	0	6	0	0	0

10	0	0	0	0	0	0	0	0	0	0
0	0	0	0	0	0	4	5	0	0	0
3	0	0	0	0	0	0	0	0	0	0
0	0	0	0	0	0	1	0	0	0	0
6	0	0	0	0	0	3	0	0	0	1
1	0	0	0	0	0	2	2	0	0	0
9	0	0	0	0	0	1	14	0	0	0
2	0	0	0	0	0	3	14	0	0	0
5	0	0	0	0	0	0	4	0	0	0
2	0	0	0	0	0	2	13	0	0	0
16	0	0	0	0	0	1	0	0	0	0
0	0	0	0	0	0	3	6	0	0	0
0	0	0	0	1	0	0	0	0	0	0
6	0	0	0	0	0	3	0	0	0	0
3	0	0	0	0	0	1	0	0	0	0
1	0	0	0	0	0	0	3	0	0	0
1	0	0	0	0	0	0	0	0	0	0
0	0	0	0	0	0	1	0	0	0	0

sp142	sp143	sp144	sp145	sp146	sp147	sp148	sp149	sp150	sp151	sp152
0	0	0	0	0	0	0	0	0	0	2
0	0	0	0	0	0	0	1	0	0	0
0	3	0	0	0	0	0	0	0	0	0
0	0	0	0	0	0	0	1	0	0	0
0	0	0	0	0	0	0	0	0	0	0
0	0	0	0	0	0	0	0	0	0	0
0	3	0	0	0	0	0	1	0	0	2
0	0	0	0	0	0	0	2	0	0	0
0	0	0	0	0	0	0	0	0	0	2
0	0	0	0	0	0	0	0	0	0	0
0	0	0	0	0	0	0	3	0	0	0
0	0	0	0	0	0	0	0	0	2	2
0	3	0	0	0	0	0	0	0	0	0
0	5	0	0	0	0	0	1	0	0	0
0	4	0	0	0	0	0	1	0	0	0
0	2	0	0	0	0	0	0	0	1	0
0	0	0	0	0	0	0	0	0	0	0
1	1	0	0	0	1	0	0	0	0	0
0	0	0	0	0	1	0	0	0	0	0
0	6	0	0	0	0	0	0	0	0	0
2	3	0	0	0	0	0	1	0	0	0
0	3	0	0	0	0	0	1	0	0	0
0	0	0	0	0	0	0	0	0	0	7
0	0	0	1	0	0	0	0	0	0	4
0	0	0	0	0	0	0	0	0	0	3
0	0	0	0	0	0	0	1	0	0	9
0	0	0	0	0	0	0	0	0	0	2
0	0	0	0	0	0	0	0	0	0	0
0	0	0	0	0	0	0	0	0	0	0
0	0	0	0	0	0	0	0	0	0	0
0	2	0	0	0	0	0	0	0	0	7
0	0	0	0	0	0	0	0	3	1	1
0	3	0	0	0	0	0	2	0	0	3
0	1	0	0	0	0	0	1	2	1	0
0	0	0	0	0	0	0	0	0	0	9
0	0	1	0	0	0	0	0	0	0	1
0	0	0	0	0	0	0	4	0	1	3
0	0	0	0	0	0	0	0	0	0	1
0	1	0	0	0	0	0	0	0	0	0
0	0	0	0	0	0	0	0	0	2	0
0	2	0	0	0	0	0	0	0	0	0
0	2	0	0	0	0	0	0	0	4	0
0	0	0	0	0	0	0	0	0	0	0
0	0	0	0	0	0	0	0	0	0	0
0	0	0	0	0	0	1	0	0	0	0
0	1	0	0	0	0	0	0	0	0	0
0	3	0	0	0	5	0	0	0	0	0
0	5	0	0	8	0	0	0	0	0	0
0	7	0	0	0	0	0	0	0	0	0
0	3	0	0	0	4	0	0	0	0	0
0	0	0	0	0	0	0	0	1	1	0

0	0	0	0	0	0	0	1	0	1	0
0	1	0	0	0	0	0	1	0	2	0
0	0	0	0	0	0	0	0	0	0	0
0	1	0	0	0	0	0	0	0	0	0
0	3	0	0	0	0	0	0	0	0	0
0	7	0	0	0	0	0	0	0	0	0
0	0	0	0	0	0	0	0	0	0	0
0	0	0	0	0	0	0	0	0	0	0
0	2	0	0	0	0	0	0	0	0	0
0	0	0	0	0	0	0	0	0	0	0
0	5	0	0	0	0	0	0	0	0	0
0	4	0	0	0	0	0	0	0	0	0
0	6	0	0	0	0	0	0	0	0	0
0	0	0	0	0	0	0	0	0	0	0
0	2	0	0	0	0	0	0	0	0	0
0	0	0	0	0	0	0	0	0	0	0
0	4	0	0	0	0	0	0	0	0	0
0	0	0	0	0	0	0	0	0	0	0

sp153	sp154	sp155	sp156	sp157	sp158	sp159	sp160	sp161	sp162	sp163
0	0	0	0	0	0	0	2	3	1	4
0	0	0	0	0	0	0	0	32	0	2
0	0	0	0	0	0	0	0	24	1	8
1	0	0	0	0	0	0	0	13	0	4
0	0	0	0	0	0	0	3	1	0	13
0	0	0	0	0	0	0	0	18	1	1
0	0	0	0	0	1	0	3	2	0	2
0	0	0	0	0	0	0	0	7	0	0
0	0	0	0	0	1	0	3	2	1	1
0	0	0	0	0	0	0	0	18	1	1
0	0	0	0	0	0	0	1	8	1	0
0	0	0	0	0	0	0	1	0	0	4
0	0	0	0	0	0	0	6	16	1	7
3	0	0	0	0	0	0	0	24	1	2
2	0	0	0	0	0	0	0	12	2	8
4	0	0	0	0	0	0	0	19	1	6
0	0	0	0	0	0	0	2	11	2	3
3	0	0	0	0	1	0	0	20	3	5
0	1	0	0	0	1	0	0	5	2	5
1	0	0	0	0	0	0	0	13	2	2
3	0	0	0	0	6	0	0	35	8	18
2	0	0	0	0	0	0	0	20	6	10
0	0	0	0	0	0	0	0	2	0	4
0	0	0	0	0	0	0	2	0	1	4
0	0	0	0	0	0	0	3	7	0	1
0	0	0	0	0	1	0	2	2	0	0
1	0	0	5	0	0	0	2	4	0	10
0	0	0	1	0	0	0	1	1	0	0
0	0	0	1	0	0	0	0	1	0	1
0	0	0	0	0	0	0	1	2	0	0
0	0	0	0	0	0	0	4	5	3	14
1	0	0	0	0	0	0	4	0	0	7
0	0	0	0	0	0	0	2	4	5	14
0	0	0	0	0	1	0	2	0	0	8
3	0	0	2	0	0	0	0	4	4	4
0	0	0	0	0	0	0	2	13	2	6
1	0	0	0	0	1	0	1	3	3	9
0	0	0	0	0	1	0	0	5	1	6
4	0	0	0	0	0	0	0	1	2	10
1	0	0	1	2	0	0	3	17	0	17
1	0	0	0	0	0	0	0	13	0	14
3	0	2	1	2	2	0	0	15	0	8
0	0	0	0	0	0	0	1	1	10	12
0	0	0	0	0	1	1	0	7	0	4
0	0	0	0	0	0	0	1	4	3	5
1	0	0	0	0	0	0	2	7	0	11
0	0	0	0	0	0	0	0	0	0	4
0	0	0	0	0	0	4	0	8	0	9
0	0	0	5	0	0	0	0	0	0	4
0	0	0	0	0	0	0	2	4	3	26
1	0	0	0	0	0	0	0	6	0	8

0	0	0	0	0	0	1	0	8	0	8
1	0	0	0	0	0	0	0	3	0	6
0	0	0	0	0	0	0	0	0	3	7
0	0	0	0	0	0	0	1	0	0	0
0	0	0	0	0	0	0	0	12	0	23
0	0	0	0	0	0	0	0	7	0	7
5	0	0	0	0	0	0	1	16	6	5
3	0	0	0	0	0	0	1	22	1	3
6	0	0	0	0	0	0	0	6	0	10
0	0	0	0	0	0	0	0	10	1	10
0	0	0	0	0	0	0	0	9	4	17
1	0	0	0	0	0	0	0	17	1	4
1	0	0	0	0	0	0	2	14	1	9
2	0	0	0	0	0	0	0	11	0	1
2	0	0	0	0	0	0	0	8	8	9
1	0	0	0	0	0	0	0	14	0	5
2	0	0	0	0	0	0	1	7	0	8
0	0	0	0	0	0	0	0	5	0	1

sp164	sp165	sp166	sp167	sp168	sp169	sp170	sp171	sp172	sp173	sp174
0	0	0	0	0	0	0	0	0	0	0
0	1	0	0	2	0	0	0	0	1	0
0	1	0	0	0	0	0	0	3	0	0
0	0	0	0	0	0	0	0	0	0	0
0	0	0	0	0	0	0	0	1	0	0
0	0	0	0	0	0	0	0	2	7	0
0	0	0	0	0	0	0	0	2	0	0
0	0	0	0	0	0	0	0	3	0	0
0	0	0	0	0	0	0	0	0	0	0
0	0	0	0	0	0	0	0	2	7	0
0	0	0	0	0	0	0	0	1	0	0
0	0	0	0	0	0	0	0	0	16	0
0	0	0	0	0	2	0	0	1	0	0
0	0	0	0	0	0	0	0	3	0	0
0	0	0	0	0	0	0	0	0	0	0
0	0	0	0	0	0	0	0	0	0	0
0	0	1	0	3	0	0	0	1	0	0
0	0	0	0	1	0	3	0	4	1	0
0	0	0	0	0	0	0	0	0	0	0
0	0	1	0	0	0	0	0	2	0	0
0	1	0	0	0	0	0	0	3	2	0
0	0	0	0	0	0	0	0	2	0	0
0	0	0	0	0	0	0	0	0	0	0
0	0	0	0	0	0	1	0	1	4	0
0	0	0	0	0	0	1	0	1	0	0
0	0	0	0	0	0	0	0	1	0	0
0	0	0	0	1	0	0	0	0	0	0
0	0	0	0	4	0	3	0	1	6	0
3	4	0	0	1	0	0	0	0	0	0
0	0	0	0	0	0	0	0	0	1	0
0	0	0	0	0	0	0	0	0	0	0
0	0	0	0	1	0	6	0	2	5	0
0	7	0	0	1	0	0	2	1	0	0
0	3	0	0	0	1	1	0	1	10	1
0	0	0	0	0	0	0	0	5	0	0
0	1	0	1	0	0	1	0	1	0	0
0	1	0	0	1	1	0	0	0	0	0
0	0	0	0	1	0	0	0	0	7	0
0	0	0	0	2	0	0	0	1	0	0
0	0	0	0	10	2	0	0	6	2	0
1	0	0	0	2	1	0	0	3	0	0
1	0	2	0	12	0	0	0	5	15	0
0	0	0	0	0	0	0	0	0	0	0
0	0	0	0	0	0	0	0	0	0	53
0	0	0	0	1	0	2	0	0	0	0
0	0	2	0	0	0	0	0	1	0	0
0	5	1	2	3	0	0	0	1	9	0
0	7	3	0	2	0	0	0	2	4	0
0	5	0	0	3	0	0	0	0	22	0
0	0	2	0	0	0	0	0	2	0	0
0	2	0	1	3	0	1	0	0	12	0

0	6	0	0	1	0	10	0	2	0	0
0	8	1	0	9	0	0	0	0	1	0
0	0	1	0	0	0	0	0	1	0	0
0	8	0	0	2	0	1	0	0	1	0
0	12	0	0	4	0	0	0	2	4	0
0	4	2	0	2	0	0	0	1	17	0
0	0	1	0	0	0	0	0	1	0	0
0	0	0	0	0	0	0	0	6	0	0
0	0	0	0	1	0	0	0	2	0	0
0	0	0	0	0	0	0	0	2	2	0
0	0	1	0	7	0	0	0	0	0	0
0	4	2	0	18	0	0	0	4	6	0
2	3	3	0	22	0	0	0	2	0	4
1	0	1	0	10	0	0	0	2	9	0
0	1	1	0	5	0	0	0	0	0	0
0	3	0	0	10	0	0	0	2	5	0
1	1	0	0	5	0	0	0	0	0	0
1	6	0	0	13	0	0	0	0	23	0

sp175	sp176	sp177							
0	0	0							
2	0	0							
0	0	0							
0	0	0							
0	0	0							
0	0	0							
0	2	0							
0	0	0							
0	0	0							
0	0	0							
0	22	0							
0	0	0							
0	0	0							
1	0	0							
0	0	0							
0	0	0							
0	0	0							
0	0	0							
0	0	0							
0	0	0							
3	0	1							
1	0	0							
0	0	0							
0	0	1							
0	0	1							
0	0	0							
0	0	0							
0	4	1							
0	0	0							
0	0	0							
0	0	0							
3	10	0							
3	0	0							
0	0	0							
0	0	0							
0	0	0							
0	0	0							
0	0	0							
0	0	0							
0	0	0							
1	0	0							
1	6	0							
3	0	0							
2	0	0							
1	0	0							
4	0	0							
0	0	0							
2	0	0							
2	0	0							
0	0	0							
2	0	0							
0	10	0							

1	0	0							
0	0	0							
2	0	0							
0	0	0							
2	0	0							
0	0	0							
3	0	0							
0	0	0							
0	0	0							
0	0	0							
2	0	0							
9	0	0							
8	0	0							
6	0	0							
3	0	0							
3	1	0							
3	1	0							
0	0	0							

